# Pharmacological impact of microRNAs in head and neck squamous cell carcinoma: Prevailing insights on molecular pathways, diagnosis, and nanomedicine treatment

**DOI:** 10.3389/fphar.2023.1174330

**Published:** 2023-05-03

**Authors:** Bedanta Bhattacharjee, Ayesha Farhana Syeda, Damanbhalang Rynjah, Shalam M. Hussain, Shekhar Chandra Bora, Padmanath Pegu, Ram Kumar Sahu, Jiyauddin Khan

**Affiliations:** ^1^ Girijananda Chowdhury Institute of Pharmaceutical Science, Tezpur, India; ^2^ Department of Pharmaceutics, Unaiza College of Pharmacy, Qassim University, Unaizah, Saudi Arabia; ^3^ Department of Clinical Pharmacy, College of Nursing and Health Sciences, Al-Rayyan Medical College, Madinah, Saudi Arabia; ^4^ Department of Pharmaceutical Sciences, Hemvati Nandan Bahuguna Garhwal University (A Central University), Chauras Campus, Tehri Garhwal, Uttarakhand, India; ^5^ School of Pharmacy, Management and Science University, Shah Alam, Malaysia

**Keywords:** head and neck squamous cell carcinoma, microRNA, molecular signaling, nanomedicine, nanovaccines

## Abstract

Head and neck squamous cell carcinoma is a disease that most commonly produce tumours from the lining of the epithelial cells of the lips, larynx, nasopharynx, mouth, or oro-pharynx. It is one of the most deadly forms of cancer. About one to two percent of all neo-plasm-related deaths are attributed to head and neck squamous cell carcinoma, which is responsible for about six percent of all cancers. MicroRNAs play a critical role in cell proliferation, differentiation, tumorigenesis, stress response, triggering apoptosis, and other physiological process. MicroRNAs regulate gene expression and provide new diagnostic, prognostic, and therapeutic options for head and neck squamous cell carcinoma. In this work, the role of molecular signaling pathways related to head and neck squamous cell carcinoma is emphasized. We also provide an overview of MicroRNA downregulation and overexpression and its role as a diagnostic and prognostic marker in head and neck squamous cell carcinoma. In recent years, MicroRNA nano-based therapies for head and neck squamous cell carcinoma have been explored. In addition, nanotechnology-based alternatives have been discussed as a promising strategy in exploring therapeutic paradigms aimed at improving the efficacy of conventional cytotoxic chemotherapeutic agents against head and neck squamous cell carcinoma and attenuating their cytotoxicity. This article also provides information on ongoing and recently completed clinical trials for therapies based on nanotechnology.

## Graphical Abstract

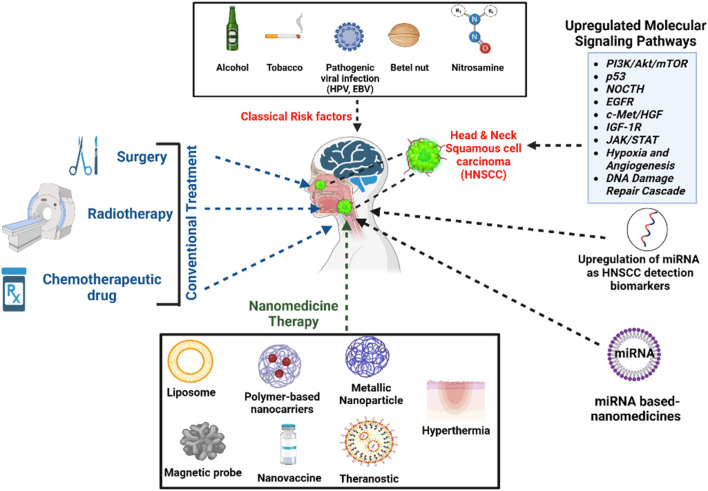

## 1 Introduction

Cancer is characterized by abnormal cell proliferation and the ability to migrate or invade other parts of the body. With the millions of cells that make up the human body, cancer can arise almost anywhere. The level of human civilization, which includes factors such as a growing and aging population, changing lifestyles, money concerns, and social and political advances, is a major cause of the increasing prevalence of cancer worldwide. In India, the projected frequency of cancer occurrences for the year 2022 is 14,61,427 (Mortality ratio:100.4/100,000) (Ertin and Kurt, 2022). Head and neck squamous cell carcinomas (HNSCCs), a complicated and multifaceted disease involving a variety of cancer cells, is one of the leading causes of cancer-related mortality worldwide (Sung et al., 2021). It is also the leading cause of morbidity and mortality worldwide. Even in developed countries, cancer is the leading cause of mortality, and in underdeveloped countries it is the second leading cause (Sung et al., 2021; Weizman et al., 2021).

HNSCCs represent heterogeneous diseases that most commonly develop tumors from the lining of epithelial cells of the lips, larynx, nasopharynx, mouth, or oropharynx (in 85% of cases). Each of these tumors has its own-epidemiology, etiology, and treatment strategy. About 6% of all cancers are HNC, which accounts for 1%–2% of neoplastic-related fatalities (Hashim et al., 2019). Additionally, squamous cell carcinomas (SCCs), which develop from the mucosal lining, account for 90% of all HNCs. In 2018, HNSCCs were the sixth most prevalent malignancy globally with 890,000 reported incidences and 450,000 deaths. The Global Cancer Observatory estimates that the number of HNSCCs will increase by 30% by 2030 (Sung et al., 2021).

HNSCCs are more likely if someone consumes areca nuts, drinks too much alcohol, uses smokeless tobacco, has a family history of the disease, or is infected with human papillomavirus (HPV) (Gislon et al., 2022). People who have never smoked continuously but have been exposed to tobacco smoke in their environment (passive smokers) have a much higher risk of developing HNSCC. The main reason for this higher rate is that carcinogens in tobacco smoke, like polycyclic hydrocarbons and nitrosamines, can cause mutations (Bukovszky et al., 2022). In recent years, smoking has declined rapidly in high-income countries, leading to a sharp decrease in HNSCCs directly associated with smoking (Fitzmaurice et al., 2017; Mourad et al., 2017). Oropharyngeal HNSCC is caused by HPV, particularly the potentially carcinogenic variant HPV16. Genetic DNA from HPV16 and 18 are detected in 25% of cases of HNSCC (Matthews et al., 2022). HNSCCs affect adults, with the average age at detection being 50 years for HNSCCs that are Epstein-Barr virus (EBV) positive, 53 years for HNSCCs that are HPV positive, and 66 years for HNSCCs that are HPV negative (Huang et al., 2015; Fung et al., 2016; Windon et al., 2018). Underlying inherited abnormalities are increasingly prone to cause HNSCC: Li-Fraumeni syndrome, Bloom syndrome, ataxia, Fanconi anemia, and telangiectasia (Subash et al., 2022). Regardless of viral or environmental causes, men are much more likely to develop HNSCC than women. The typical symptoms of HNSCC depend on where the original tumor is pinpointed in the body and what causes it to grow (environmental carcinogens, HPV, or EBV) (Huang et al., 2015). A non-healing oral ulcer or irritation is the traditional presentation of HNSCC. HNSCC can cause odynophagia, which is a pain in swallowing, otalgia, which is a pain in the ear, and dysphagia, which is difficulty swallowing (Florido et al., 2022). Patients with HNSCC may have respiratory problems that can lead to airway obstruction and require tracheotomy if the tumors are not treated (Caudell et al., 2022; Goyal et al., 2022).

Depending on the grade of tumor nodal metastasis (TNM) and where the cancer started, HNSCC is treated in different ways (Caudell et al., 2022; Tawk et al., 2022). These include resection, radiotherapy (intensity-modulated radiotherapy or external beam radiotherapy), and conventional cytotoxic chemotherapeutic agents (cisplatin, docetaxel, pembrolizumab, or cetuximab). Approximately 40% of patients have early-stage disease (stage I or II), which is often treated with resection or radiotherapy alone (Caudell et al., 2022). In the majority of patients with regionally advanced malignancy (stage III and IVA/B), therapy includes platinum-based chemoradiation. In general, chemotherapeutic agents and radiotherapy are associated with nausea, dry mouth, loss of taste sensation, hair loss, sore throat, dry skin, and cytotoxicity, which to some extent causes severe organ damage (Caudell et al., 2022; Tawk et al., 2022).

When cancer is detected early, it can be treated more successfully, improving the outlook of the disease and reducing its impact on other tissues and organs. The use of radiation therapy and chemotherapeutic agents as adjuvant systemic treatments are two common components of standard cancer therapy regimens. The inability of these anticancer drugs to differentiate between healthy normal cells and cancer cells results in adverse effects as well as low levels of the drugs reaching the desired target (low bioavailability), susceptibility to enzymatic degradation, reduced half-life of the drug in the systemic circulation, impaired cellular uptake, and upregulation of p-glycoprotein, which compromises curative efficacy. Therefore, there is a need to develop novel formulations that can overcome all the limitations of conventional cancer treatment.

Currently, nanomedicine has become a new strategy in cancer treatment. Nanomaterials such as metal-organic frameworks (MOFs), micelles, multistage vector (MSV) platforms, and liposomes have been optimized as nanomedicines to produce targeted therapeutic delivery platforms (Wong et al., 2020). An important point to think about is how to make targeted nanomedicines to deliver microRNAs (miRNAs), chemotherapeutics, and tumor-associated antigens into cancer cells more efficiently and effectively, improve bioavailability, redesign the immune system, and reduce immune-related side effects and cytotoxicity (Goldberg, 2019). miRNA-based nanomedicine therapy can replace conventional therapies by overcoming the shortcomings of existing treatments such as reducing multidrug resistance, cell death, and non-specific distribution errors while attempting to maximize the efficacy of therapeutic drugs. These manufactured nanomedicines can efficiently overcome various barriers such as biological membranes, blood capillaries, and cellular barriers (Gharat et al., 2016; Zhao et al., 2017; Bharadwaj and Medhi, 2020). In this review, we aim to highlight the molecular signaling cascades associated with HNSCCs. We also provide an overview of miRNA down- and upregulation, its role as a prognostic marker and biomarker in HNSCC, and the treatment of HNSCC with nano-based therapeutics. In addition, nanotechnology alternatives are discussed as a promising strategy for exploring therapeutic paradigms aimed at improving the efficacy of traditional cytotoxic chemotherapeutics against HNSCCs and reducing their cytotoxicity.

## 2 Molecular signaling cascades associated with HNSCC

### 2.1 PI3K/Akt/mTOR pathway

**FIGURE 1:**
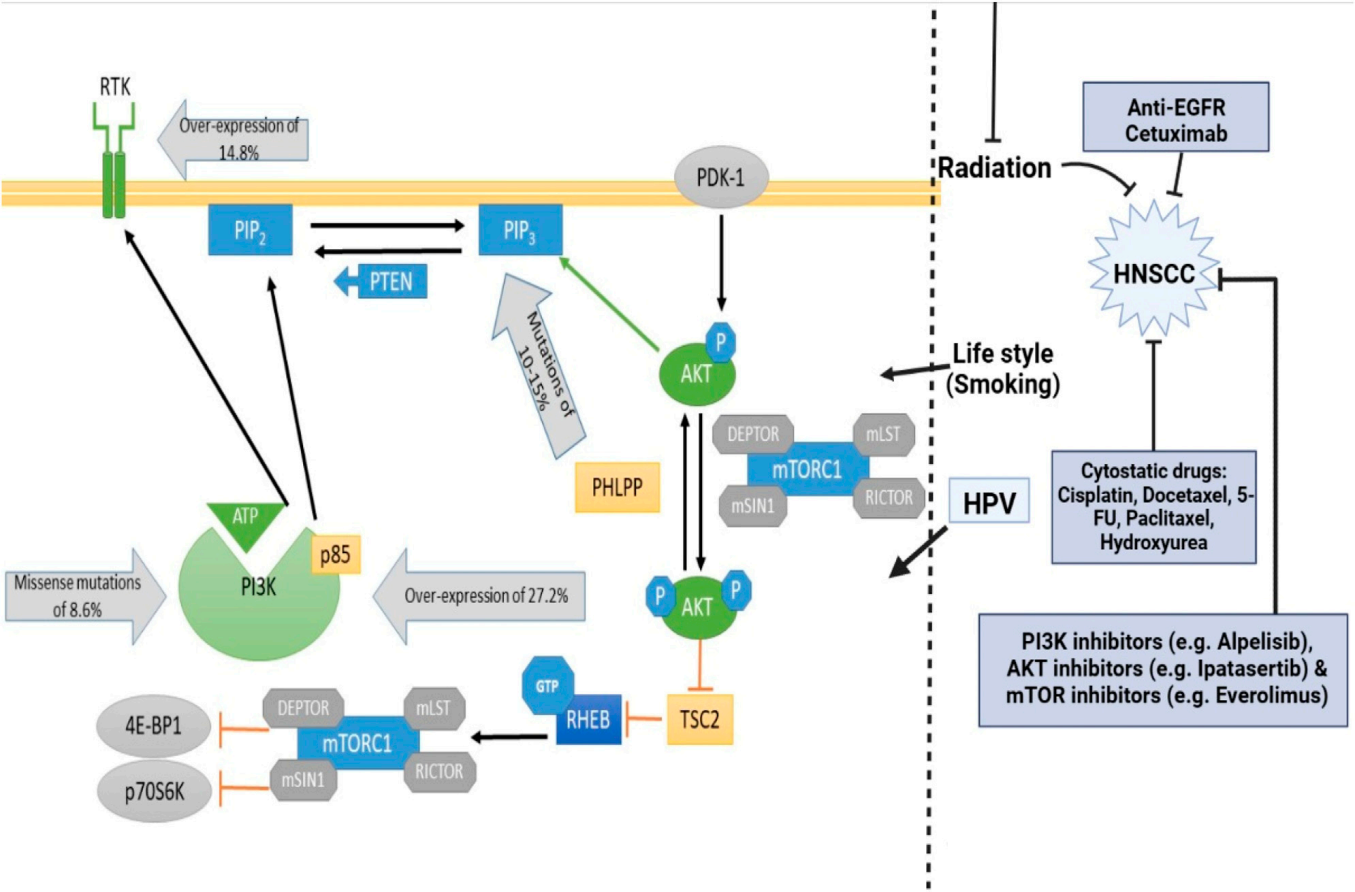
Phosphylation of domain 3 OH of PIP2 by the regulatory component leads to the formation of PIP3 upon modulation by EGFR. PIP3 then recruits proteins with pleckstrin homology motifs (PH) to the cell membrane, specifically Akt and phosphoinositide-dependent protein kinase 1 (PDK1), causing PDK1 and Mammalian Target of Rapamycin Complex 2 to phosphorylate AKT. AKT is triggered and consequently, mTORC1 is activated, which in turn stimulates P70S6 kinase and inactivates eukaryotic translation inhibitory factor 4E-binding protein 1, inducing cellular survival, cellular proliferation, and protein biosynthesis. The tumor suppressor genes PTEN modulate the intracellular concentration of PIP3 by converting it to PIP2 via its lipid phosphatase ability, thus preventing the initiation of Akt and its cascade networks.

The PI3K (phosphatidylinositol 3-kinase)/Akt (protein kinase B)/mTOR (mammalian target of rapamycin) regulatory cascade is one of the downstream modulatory circuits of epidermal growth factor receptor (EGFR) and is important for mediating physiological regulated events such as cell expansion, division, longevity, proliferation, migration, glycolysis, angiogenesis, apoptosis, and cell metabolism (Fruman et al., 2017; Ali et al., 2022). Based on their major sequence, coding, and lipid stereoselectivity, PI3K protein kinases and lipids are categorized into three classes: Class I, II, and III. But only class I protein kinases work as secondary signaling molecules in cellular signaling systems, and they are most often linked to the growth of tumors. The main components of class I PI3K, which are 85 kilodaltons (p85) and 110 kilodaltons (p110) and are heterodimeric, help with enzyme catalysis and the related inhibitory activities (Stanciu et al., 2022). There are three different variants of the p85 component, mainly p85α (expressed by PIK3R1 genes), p85β (expressed by PIK3R2 genes), and p58γ (expressed by PIK3R3 genes), while the p110 component is expressed by three genes, namely, PIK3CA (p110α), PIK3CB (p110β) and PIK3CD (p110γ) (Stanciu et al., 2022). Phosphorylation of domain 3 OH of PIP2 (phosphatidylinositol 4,5-bisphosphate) by the regulatory component leads to the formation of PIP3 (phosphatidylinositol 3,4,5-triphosphate) upon modulation by EGFR. PIP3 then recruits proteins with pleckstrin homology motifs (PHM) to the cell membrane, specifically Akt and phosphoinositide-dependent protein kinase 1 (PDK1), causing PDK1 and mammalian target of rapamycin complex 2 to phosphorylate Akt. Akt is triggered and consequently, mTORC1 is activated, which in turn stimulates P70S6 kinase and inactivates eukaryotic translation inhibitory factor 4E-binding protein 1, inducing cellular survival, cellular proliferation, and protein biosynthesis (Kannaiyan and Mahadevan, 2018). In addition, the tumor suppressor genes PTEN (phosphatase and tensin homolog) modulate the intracellular concentration of PIP3 by converting it to PIP2 (phosphatidylinositol 4,5-bisphosphate) v*ia* its lipid phosphatase ability, thus preventing the initiation of Akt and its cascade networks (Yang Z. et al., 2022). Four cell lines displayed Akt1 action in response to radiation and were sensitive to mTORC1 and Akt dual suppression. In order to overcome radio resistances, it may be able to both inhibit mTORC1 and Akt. High-grade tumors and malignancy are linked to serine phosphorylation-induced Akt2 action. Human keratinocytes exposed to HPV16 exhibited increased *in-vitro* Akt and mTOR activities. Moreover, the root causes the phosphorylation of 4E-BP1 and S6K, two mTOR complex 1 intermediate. These are able to encourage protein expression while suppressing apoptosis (Marquard and Jücker, 2020). Genomic alterations in the PI3K/Akt/mTOR circuit are highly conspicuous in head and neck malignancies (Figure 1) (Gougis et al., 2019). In 2011, genomic microarray results confirmed that upregulation of the circuit (in the case of HNSCCs) is favored by 6%–20% genetic variations in PI3KCA genes encoding p110α. Moreover, 80% of genetic variations unfold in exon 4, exon 20 (kinase unit), and exon 9 (helical unit), mainly *via* paradigms of genomic modulation (Lee S. Y. et al., 2022). Interestingly, PTEN alterations (phosphatase and tensin homolog) were detected in 10% of HNSCC incidences, although they may not represent the predominant pathway for PTEN depletion in HNSCC (Squarize et al., 2013).

### 2.2 Tumor protein (p53) pathway

**FIGURE 2:**
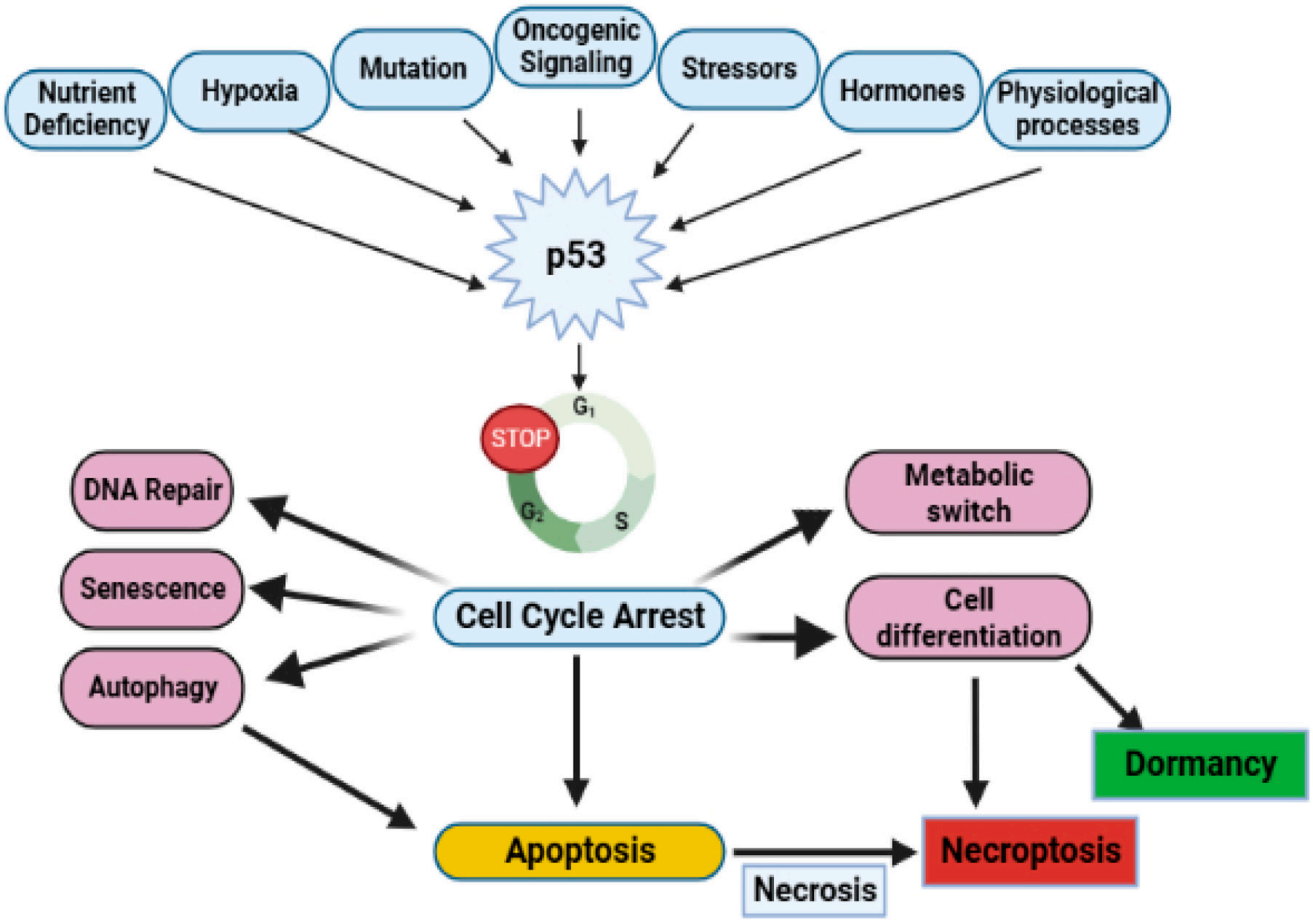
Functions of p53 in tumor suppression, both canonical and non-canonical. Numerous cellular stress signals can trigger the p53 protein. Nutrient stress, hypoxia, oncogene activation, DNA damage, and oxidative stress from reactive oxygen species (ROS) are some of the inducers of p53 that lead to a rise in p53 action. The transcriptional and translational reactions of p53 comprise cell cycle arrest and repair of DNA damage, which puts the cell in a phase of senescence or triggers apoptosis. Autophagy routes, necrosis, necroptosis, and ferroptosis are examples of non-canonical, regulated programmed cell death responsibilities. In addition, p53 functions as a switch in the metabolic mechanism implicated in differentiating, and rerouting specialized cell activity, and normal physiological processes like hormone stimulation can also culminate in p53-induced downregulation.

The tumor suppressor function of p53 was identified at the start of the 21st century. In human malignancy, p53 is the gene that is most commonly altered. The tumor suppressor p53 is successfully recognized for its functions in triggering cell cycle halt, ferroptosis, apoptosis, repair of DNA, and aging which facilitates p53 tumor inhibitory role. More than 50% of HNSCC cases have genetic variations of the tumor protein (p53), making it one of the most commonly distorted markers in HNC [32]. Cells that have lost the potential to inhibit their development and are unable to adapt to DNA malfunction or stressors are triggered by the tumor suppressor p53 being immobilized on chromosome 17q13 (Figure 2) (de Bakker et al., 2022). Approximately 50% of all human malignancies have oncogenic p53 mutations that cause alteration or loss of function, making it one of the most commonly mutated components in malignancies. A variety of physiological stress stimuli that activate p53 post-translationally cause it to stimulate the development of genes associated with cell cycle inhibition, apoptosis, cell abrogation, and DNA rescue. This occurs due to the extensive transcriptional regulatory function of p53 (Moulder et al., 2018). In parallel with aberrant p53, other genes in the p53 circuit are often disrupted or downregulated, leading to p53 impairment. The ataxia telangiectasia mutated (ATM) gene, identified on chromosome 11q22-23, can be altered and cause ataxia telangiectasia. ATM is a kinase that is triggered primarily by double-stranded DNA breaks (DSBs), thereby stimulating p53. The inactivation of ATM is a hallmark of p53 dysfunction (Perrotti et al., 2022). The p53 blocker HDM2 is bound by p14arf, which is bound to p53 and triggers p53. A common genetic occurrence in the progression of HNSCCs is a mutation in the gene ARF, which causes the appearance of p14arf (Chantre-Justino et al., 2022). In addition to p53 signaling pathways, secondary factors such as B-cell lymphoma 2 associated X protein (Bax) and Bcl-2 of the apoptotic regulator may be affected (Rajabi-Moghaddam and Abbaszadeh, 2022). A prognostic marker for oropharyngeal HNSCCs is human papillomavirus type 16 (HPV16) (Boza. 1999) p53 is inactivated by E6, a viral oncoprotein of HPV16, through enzymatic activity (Chung and Gillison, 2009).

### 2.3 Neurogenic locus notch homolog protein (NOCTH) signaling pathway

**FIGURE 3:**
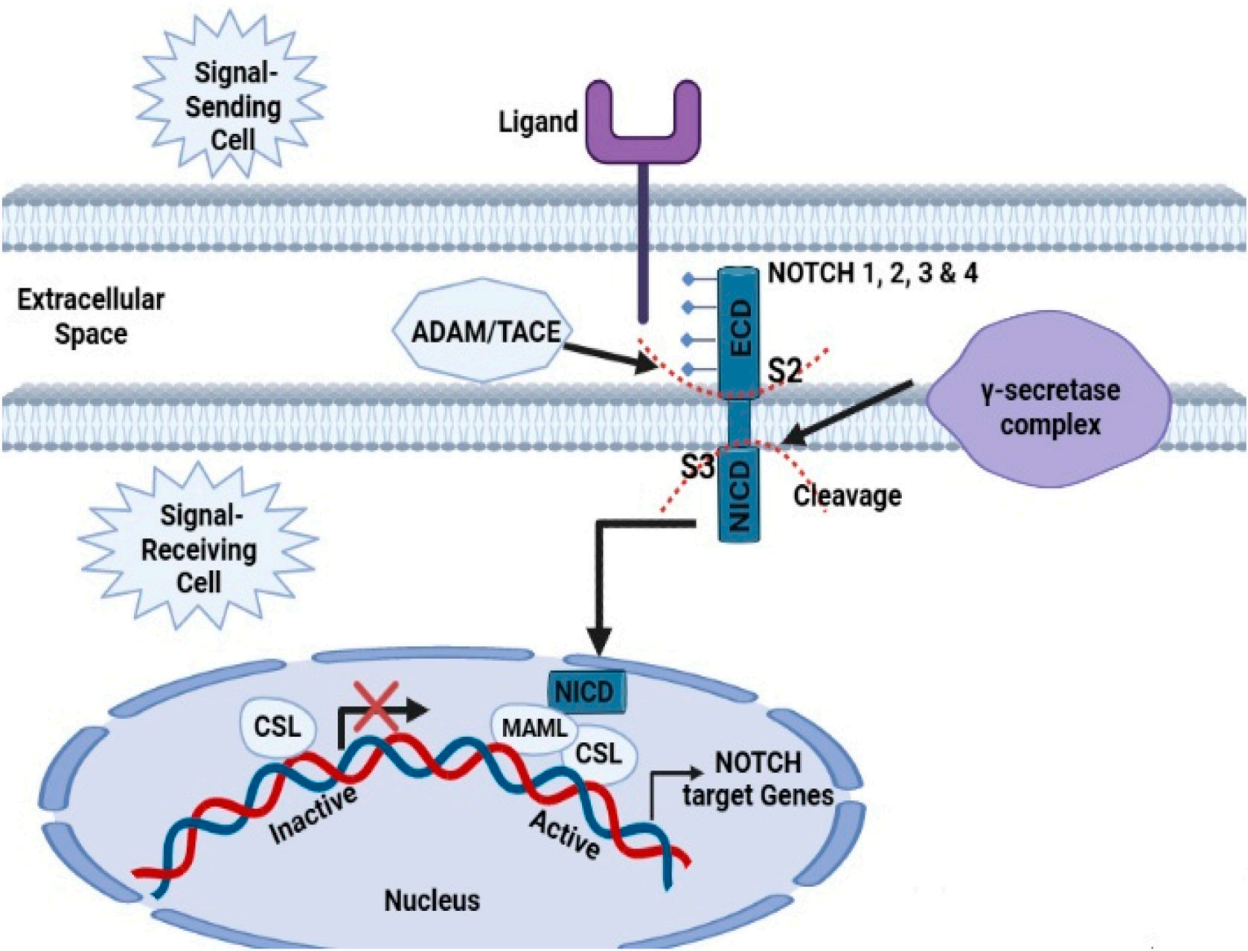
Activation of the primary NOTCH1 signal. When a ligand is bound, two subsequent proteolytic cleavages start (S2 and S3). The initial cleavage at the S2 location of the extracellular domain is mediated by the ADAM/TACE proteinase. Accessibility to the γ-secretase complex, which is in charge of the subsequent proteolytic cleavage at the S3 site inside the transmembrane region, is made possible by the S2 cleavage. When the NICD domain is freed, it moves to the nucleus and engages with the CSL transcription factor. The activation of mastermind-like protein (MAML) is facilitated by the establishment of a composite-binding interface by docking the NOTCH ankyrin repeat domain to the CSL protein. Through the displacement of corepressors and histone deacetylases and the recruitment of histone acetyltransferases, these processes change CSL from a transcriptional repressor to a promoter. As a result of MAML recruitment of further coactivators, the NOTCH target genes are expressed.

The NOTCH circuit system is a persistent signal transduction pathway analogous to mitogen-activated protein kinase (MAPK) and PI3K/Akt that modulates cellular properties, including the potential for cell regeneration and maturation (D’assoro et al., 2022). NOCTH signaling is an intercellular signaling process that plays multiple roles in the growth of arteries, nephron parts, and the nervous system. It is involved in the development and division of numerous cell types and can efficiently control cell fate. The NOTCH group includes 4 receptors (NOTCH1, NOCTH2, NOCTH3, and NOCTH4) that are attached to biological membranes and bind with several classes of canonical mediators, namely, Jagged (JAG1 and JAG2) and Delta-like (DLL1, DLL3, and DLL4) ligands (Tchekneva et al., 2019). When a mediator binds to the NOTCH receptor sites, γ-secretase and TNF-α converting enzyme (TACE) fragment the NOTCH protein and release the NICD, *i.e.*, NOTCH intracellular domain. The NICD consists of several motifs, such as ankyrin repeats (ANK), RAM, JM, and the transcriptional activation motif (TAM); however, TAM is deleted from NOTCH 3 and NOTCH 4 (D’assoro et al., 2022). To date, solid tumor cells have been shown to have bidirectional NOTCH signaling. Initial studies indicated that NOTCH1 alterations have a cytotoxic effect in protracted hematologic malignancies and T-cell acute lymphoblastic leukemia (Pinto et al., 2020). NOTCH1 was found to be the second most frequently mutated gene in the genomes of HNSCCs, suggesting that it functions as a tumor suppressor gene with a prevalence rate of 15%–19% (Khan and Darido, 2022). A 13-year dataset of 128 individuals with HNSCCs served as a platform for the evaluation of several NOTCH1 spontaneous mutations, which were investigated using whole-exome analysis in 2016. According to a recent holistic review, the NOTCH system is dysfunctional in 66% of individuals with HNSCC (Liu et al., 2016). The endothelial growth factor receptor system indirectly modulates NOTCH1 signaling, which promotes keratinocyte endpoint development. In epidermal squamous cell carcinomas, NOCTH1 and p53 are inhibited by EGFR-triggered c-jun, but the restriction of EGFR stimulates keratinocyte development (Gürsel Ürün, 2022). Interestingly, NOTCH1 activity in epithelial stem cells is actually inhibited by tumor protein p63 (TP63) as a transcription factor, resulting in NOTCH1 overexpression rather than dysregulation throughout cellular maturation. The proliferation and upregulation of TP63 has been demonstrated in several HNSCC incidences (Figure 3) (Capodanno et al., 2021). A gateway impeding the degradation of functional NOTCH1 is F-Box and WD repeat domain containing 7 (FBXW7) mutation in 5% of HNSCC incidences (Chan et al., 2019). Only a few research papers have specifically addressed the role of NOTCH1 activation in HNSCC, although the link between deregulation of NOTCH1 signaling and cancer in humans is clear. While the multitude of mostly suppressive variants of NOTCH1 shows that it acts as a tumor regulator, several investigators reported that activation of NOTCH can promote proliferation, prevent apoptosis, and stimulate angiogenesis, suggesting that NOTCH1 may occasionally act like an oncogene. Chromosomal mutations such as MAL1, NUMB, JAG1, and JAG2 have been shown to play a role in altering NOTCH subtleties in addition to NOTCH (1–3) abnormalities (3%–5% of cases) (Schubert et al., 2018).

### 2.4 Epidermal growth factor receptor (EGFR) pathway

**FIGURE 4:**
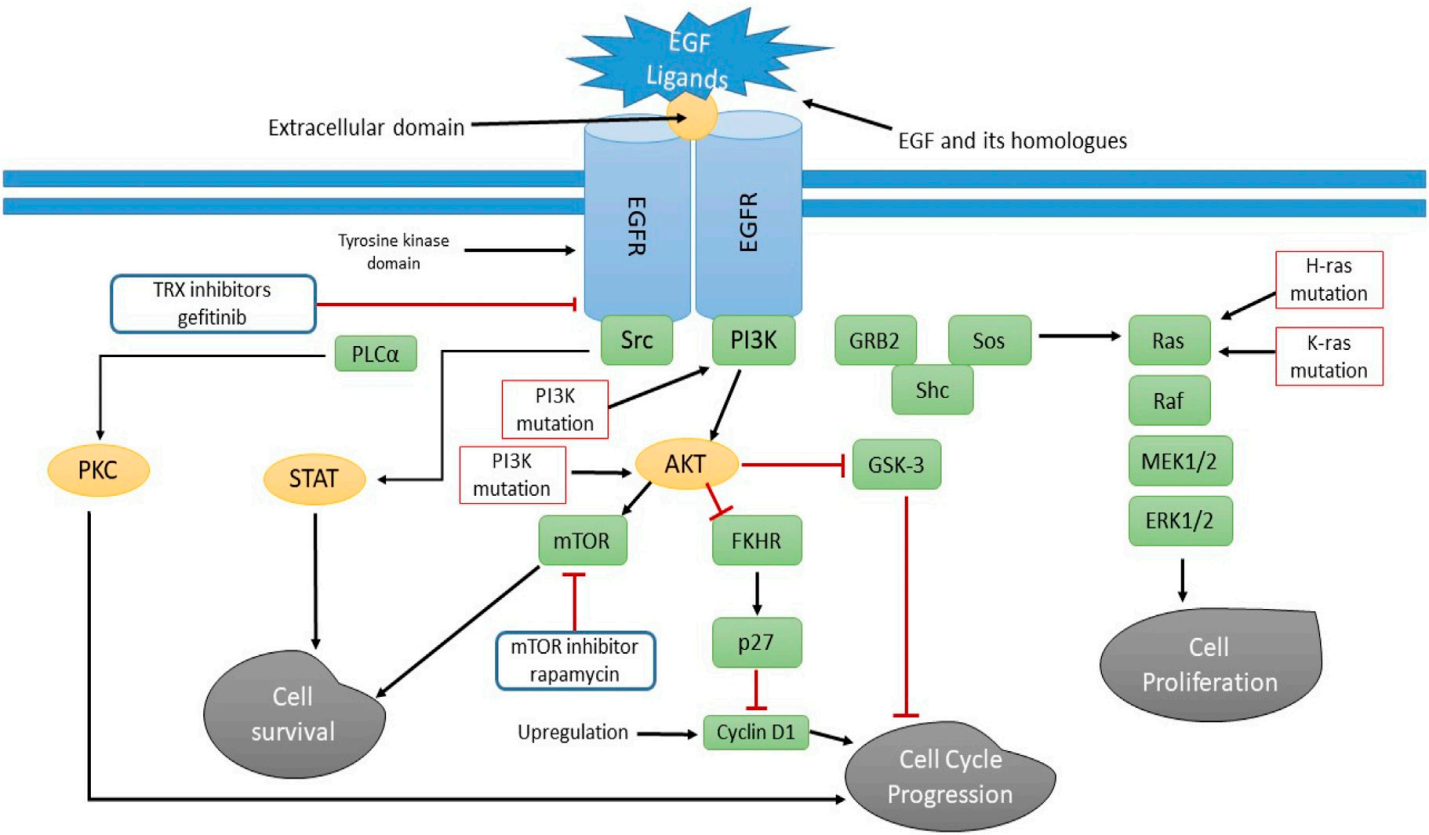
EGF (and its homologs) attachment to EFGR causes direct or indirect cascades of events that result in cell viability and cell proliferation. The main characteristics of cancer progression-increased growth, decreased apoptosis, enhanced angiogenesis, and metastasis are associated with somatic EGFR mutations that result in sustained activation. The illustration highlights the method for suppressing abnormal EGFR activation in tumor cells utilizing tyrosine kinase inhibitors.

The tyrosine kinase receptor complex, which also includes ErbB2/Neu/HER2, ErbB4/HER4, and, ErbB3/HER3 includes the epidermal growth factor receptor (EGFR/ErbB1/HER1), which is suggested to be a proto-oncogene. For EGFR to work properly, its cytoplasmic tyrosine kinase domain needs to be stimulated. To stimulate EGFR, the receptor needs to be attached to its extracellular domain (ECD). Under physiological conditions, EGFR is to control angiogenesis, cellular growth, homeostasis, and the maturation of epithelial tissues. Modulation of renal blood flow and control of electrolyte balance by the kidney under all biological conditions appears to be significantly influenced by EGFR activity. Throughout the development of cancer, tumor cells also produce comparable ligands that support autocrine and paracrine activities. Ras/Raf/MAPK, PI3K/Akt, JAK/STAT, or PLC/PKC pathways may also be activated by EGFR signaling. These pathways are implicated in a variety of cellular functions, including metabolism, growth, survival, apoptosis, and differentiation (Usman et al., 2021). Modifications in regulatory cascades of growth factors also contribute to the underlying etiopathogenesis of HNSCCs. EGFR (ErbB1 or HER1) is a transmembrane glycoprotein and belongs to a class of tyrosine kinase receptors that are abundant in mammalian epithelial cells. The EGFR regulatory mechanism is the basis for the functioning of mammalian cells. It controls cell division, programmed cell death, migration, intercellular transport, and proliferation throughout the maturation cycle. The most common way EGFR is turned up in HNSCCs is through the start of transcription and chromosomal division (Zhu et al., 2022). EGFR can be triggered by numerous signaling molecules, *viz* amphiregulin (AREG, a transmembrane glycoprotein with 252 amino acids) and transforming growth factor alpha (TGF-β, an endogenous peptide). When tyrosine kinase interacts with signaling molecules, it gets phosphorylated and forms homo- and heterodimers, which start the signal propagation networks (Mondal et al., 2022; Osude et al., 2022). The vast majority of HNSCC tumors exhibit upregulation of EGFR (Nguyen et al., 2022). Although genetic mutations in the EGFR genome have been detected in approximately 7%–30% of HNSCC patients, this suggests that most EGFR upregulation occurs in the translational domain (Bhatia, 2022). In 42% of HNSCC patients, a mutant EGFR version, *i.e.*, EGFRvIII, was observed, characterized by 2-7 exons deleted in the extracellular domain. The activated network pathways Janus kinase (JAK), PI3K/Akt/mTOR, and mitogen-activated protein kinase (MAPK)/Raf/Ras are upstream of EGFR (Figure 4) (Kang et al., 2023).

### 2.5 Mesenchymal-epithelial transition factor (c-Met)/hepatocyte growth factor (HGF) pathway

**FIGURE 5:**
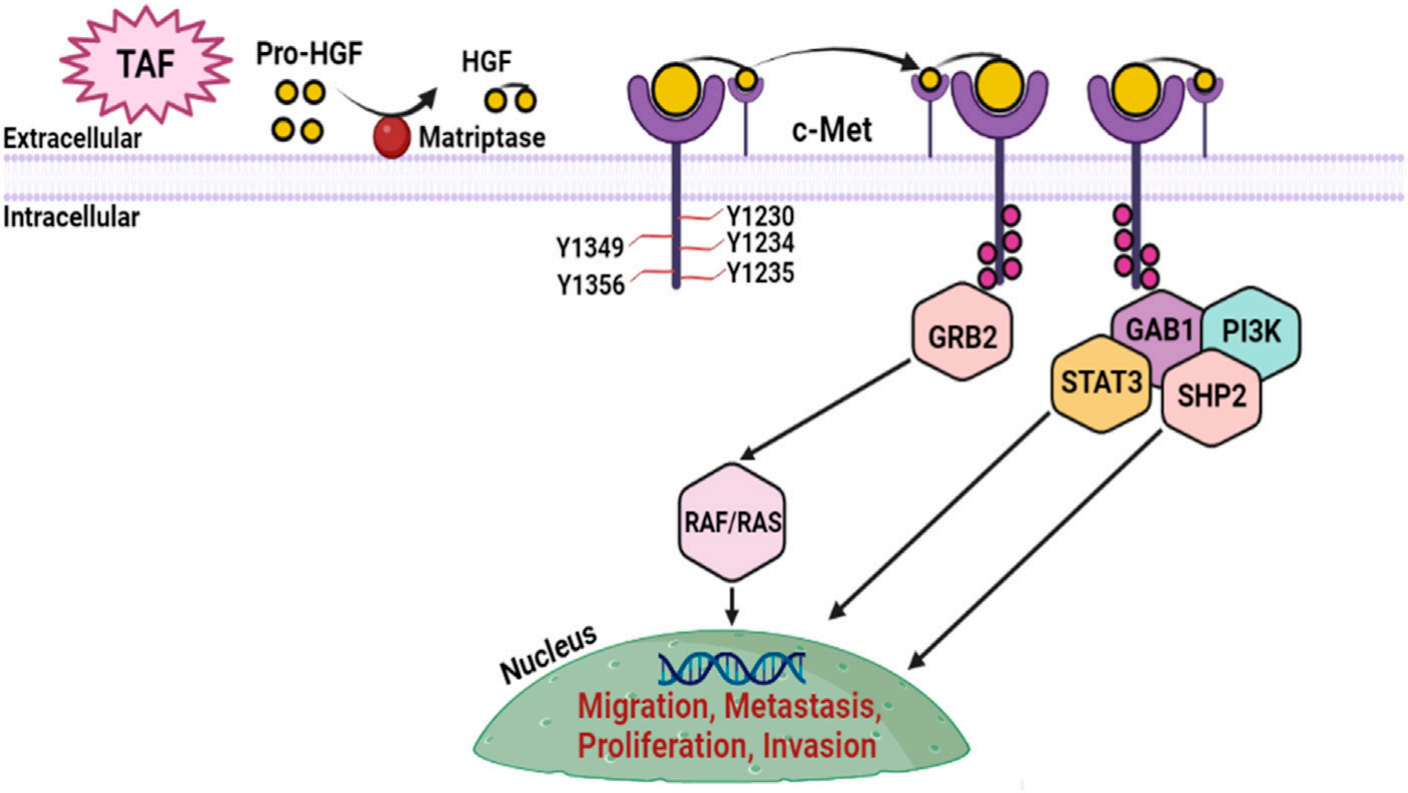
c-Met/HGF pathway in HNSCC. Pro-HGF is released by TAFs and is degraded by cell surface matriptase, allowing the dimer molecule to activate the c-Met binding site. Following activation, c-Met proceeds through phosphorylation and attracts intermediary molecules Gab1 and Grb2, which then attract oncogene molecules STAT3, Ras/Raf, PI3K, and SHP2 starting regulatory pathways that induce dissemination, motility, and infiltration.

c-Met/HGF, which is regulated by the tumor suppressor p53, can facilitate the growth of muscle and nerve, cellular motility, cellular proliferation, angiogenesis, wound recovery, tissue regeneration, tissue homeostasis, organogenesis, and the development of embryos during physiological circumstances. The Met (encrypts c-Met) proto-oncogene, which is localized on human genome 7 (7q21-q31), transcribes the protein tyrosine kinase identified as Met (Centuori and Bauman, 2022). Met is constituted of numerous structural elements, namely, the semaphorin (SEMA), the protein tyrosine kinase motif (PTKM), and the juxtamembrane (JM-operational motif), which adheres to its receptor, *i.e*., hepatocyte growth factor (HGF) (Faiella et al., 2022; Raj et al., 2022). Most of the HGF in HNSCC is expressed by tumor-associated fibroblasts (TAFs) in the tumor microenvironment as an ineffective proenzyme that must be enzymatically degraded by the cell surface protease, matriptase (Yamashita et al., 2022). Coupling of MET to HGF promotes upregulation of the enzymatic efficiency of tyrosine kinase, namely, Y1235, Y1234, and Y1230, MET dimerization, and intracellular phosphorylation, leading to cellular expansion, migration, and longevity (Zhang et al., 2018). Tyrosines Y1356 and Y1349 are consequently phosphorylated and form a chelate-binding domain that attracts and adheres to the intermediate molecules, growth factor receptor bound protein 2 associated binder 1 (Gab1), and growth factor receptor bound protein 2 (Grb2) which are necessary for intracellular HCF/c-MET regulation. Gab1 stimulation by phosphorylation triggers the upregulation of signal transducers and activators of transcription-3 (STAT3), SH2-containing protein tyrosine phosphatase (SHP2), and phosphoinositide 3-kinase (PI3K) whereas phosphorylation of Grb2 triggers the tumorigenic Raf/Ras pathway which results in tumor growth, multiplication, and migration (Figure 5) (Raj et al., 2022). Genomic complications such as Met gene duplication, Met alterations, and upregulation of either HGF protein or c-Met all lead to elevated stimulation of the c-Met/HGF system. Approximately 90% of HNSCC malignancies exhibit upregulation of the c-Met, whereas upregulation of mRNA, is also often documented (Dioguardi et al., 2022). Phosphorylation or activation of c-Met (p-Met) is frequently detected in clinical samples of HNSCC. In a study that examined protein activity patterns of malignant and healthy HNSCC tissues, the stimulatory tyrosines Y1235, Y1234, Y1230, and Y1003 of p-Met were found in 66% of malignant tumors, consistent with total c-Met transcription in 79% of malignant tumors (Faiella et al., 2022). In combination with the upregulation of p-Met and c-Met, MET alterations in SEMA, PTKM, and the JM operating motif were detected in HNSCC malignant patients. The triggering missense variation Y1235D was reported in research by Hagege *et al.* with a greater prevalence in disseminated lymphatic organs from HNSCC individuals correlate to the matching original tissue, indicating polyclonal screening of the variation and indicating that c-Met regulates dissemination (Hagege et al., 2022). 45% of initial HNSCC malignancies have upregulated HGF protein, which has been revealed in conjunction with c-Met transcription. More than 58% of RM (relapsed metastatic) HNSCC individuals had significant HGF expression of genes (Faiella et al., 2022). Phosphatidylinositol 3-kinase (PI3K) and mitogen-activated protein kinase (MAPK) are two downstream mechanisms that are triggered when c-Met/HGF signaling is downregulated and that support the advancement and viability of cancerous cells (Li et al., 2023).

### 2.6 Insulin-like growth factor-1 receptor (IGF-1R) pathway

**FIGURE 6:**
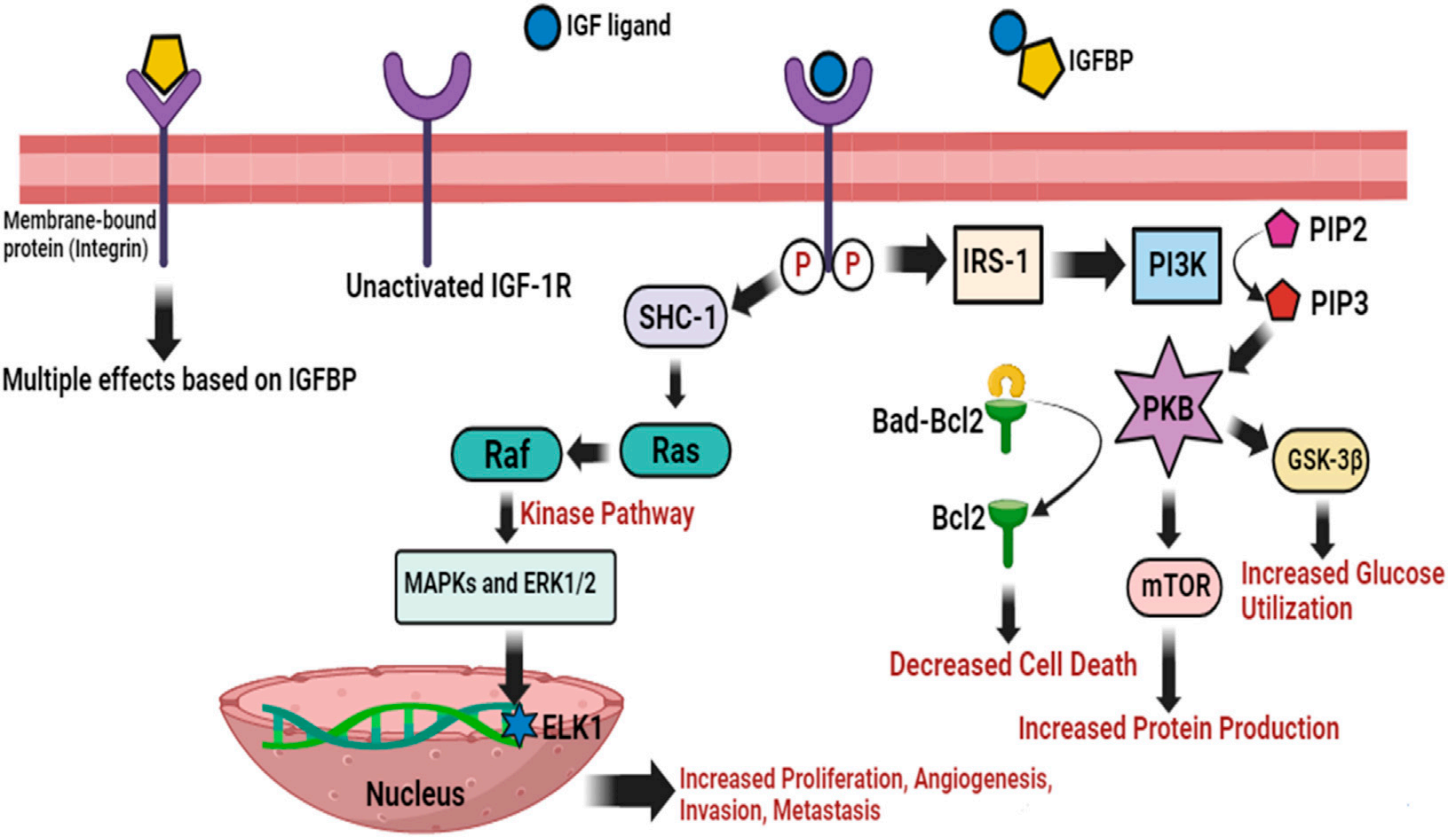
IGF-1R activation network with substantial effector molecules. PI3K/PKB and MAPK/Ras are two regulatory cascades that can be triggered by IGF-1R. Cell death, glucose utilization, and protein production are all improved by PI3K/PKB. MAPK/Ras involves a complex kinase pathway that, in response, promotes the expression of transcription factors, *i.e*., ELK1 to accelerate cellular proliferation, angiogenesis, invasion, and metastasis. IGF binding proteins (IGFBPs) regulate the action of IGF ligands by direct interaction in the outer membrane environment. IGFBPs potentially have a variety of IGF actions due to their direct interactions with integrins.

Insulin-like growth factor-1 (IGF-1) is a natural growth hormone and plays a physiological function essential for tissue growth and homeostasis, adolescent development, and fetal growth. IGF-1 effectively enhances bone structure, bone thickness, and muscle strength. IGF-1 participates in postnatal development and embryogenesis in conjunction with growth hormones. IGF-1 has physiological benefits on cellular expansion, anti-oxidative, hepatoprotective, neuroprotective, anti-aging, anabolic, anti-inflammatory, and carbohydrate and lipid metabolism properties. The proliferation and longevity of numerous tumor forms, particularly HNSCC, have been linked to the insulin-like growth factor-1 receptor (IGF-1R) and its ligands IGF-1 and IGF-2. IGF-1R is triggered by post-translational alteration when it binds the IGF ligand, and then upregulates insulin receptor substrate-1 (IRS-1) (Cao et al., 2022). PI3K is subsequently stimulated, which results in a rise in PIP3 then activating the essential protein kinase B (PKB or Akt) *via* phosphorylation (Ma G. et al., 2022). The anti-apoptotic molecule Bcl-2 is consequently released from Bad by PKB, which also promotes mTOR to initiate protein production and hinders GSK-3β to improve glucose utilization (Lee J. S. et al., 2022). It is known as the PI3K/PKB cascade of IGF-1R regulation and is primarily essential for cell death (Choi, 2022). In addition, IGF-1R promotes cell proliferation and migration, and changes in cell adherence *via* MAPK/Ras system all leading to rapid tumor growth (Mudra et al., 2021). IGF-1R promotes Raf with the Ras GTPase by triggering the SHC-transforming protein 1. Raf immediately initiates a kinase pathway, which leads to the stimulation of ERK1/2 (extracellular signal-regulated kinase), and MAPKs. A number of substrates are subsequently phosphorylated and triggered by the MAPKs, including significantly the ELK1 (function as transcription activator), which induces the expression of genes and hence cellular proliferation, angiogenesis, invasion, and metastasis (Figure 6) (Das et al., 2022). It has been shown that IGF-1R is turned up in HNSCC, and blocking it lowers PI3K and MAPK signaling pathways and makes the cells move and multiply less (Li F. et al., 2022).

### 2.7 Janus kinase (JAK)/signal transducer and activators of transcription (STAT) cascade

**FIGURE 7:**
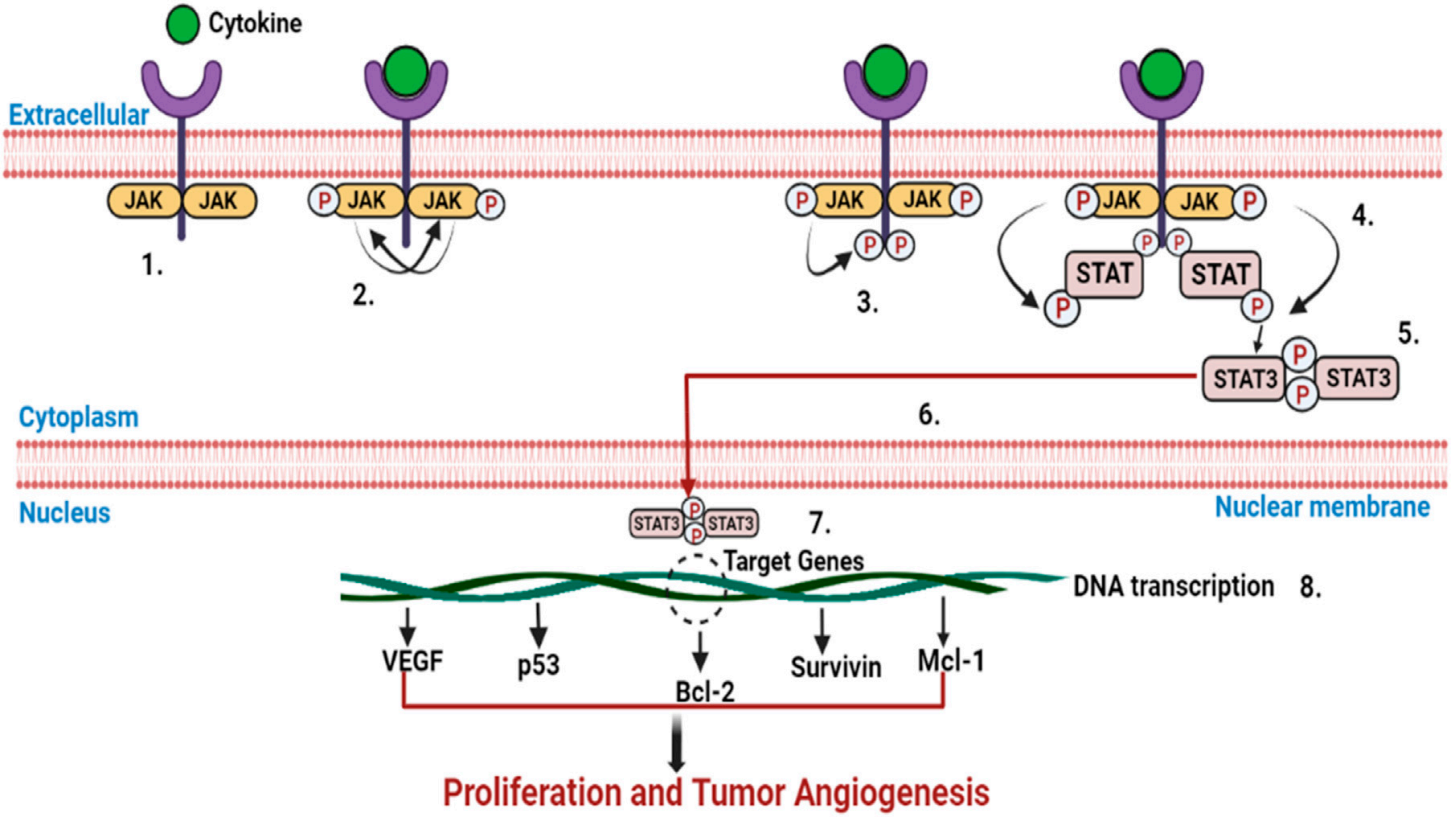
Infographic depicting how stimulation of the JAK/STAT signaling cascade induces the overexpression of genes associated with proliferation and tumor angiogenesis. The JAK/STAT pathways go through a number of cycles: 1: Cytokines adhere to receptors, which further crosslink. 2: Phosphorylation of JAKs on one another. 3: Phosphorylation of the receptor through JAKs, creating phosphotyrosine interacting regions for STAT SH2 motif. 4: STAT interacts with the receptor. Phosphorylation of the STAT *via* JAK changes the structure of STAT. 5: The dissociation of STAT phosphorylation from the receptor and cross-linking. 6: The STAT phosphorylation immediately moves inside the nucleus. 7: STAT phosphorylation interacts with DNA. 8: Activation of DNA transcription of target genes.

The regulatory cascade (JAK/STAT) transmits commands from cell membranes to cell nuclei and regulates actions such as cell development, proliferation, division, longevity, angiogenesis, and immunological responses. The JAK/STAT system controls activities including lactation, hematopoiesis, development of mammary glands, inflammatory response, stem cell repair as well as embryonic maturation. The dissociation of STAT from the receptor and the crosslinking of 2 STAT subunits occur as a response to the phosphorylation of STAT on its residual tyrosine domains during receptor pair stimulation. The crosslinker immediately moves inside the nucleus, binds to DNA there, and activates the production of a cytokine-responsive gene (Figure 7) (Philips et al., 2022). The JAK group of intracellular non-receptor tyrosine kinases includes 4 enzymes, namely, tyrosine kinase 2 (TYK2), JAK1, JAK2, and JAK3, all of which comprise 7 parts called Janus homologous motifs 1–7 (JH1-7) (Liu et al., 2023). The STAT cytoplasmic proteins are a group of intracellular transcription factors with 7 known components, namely, STAT1, STAT2, STAT3, STAT4, STAT5A, STAT5B, and STAT6. Each component of the STAT family has 6 homologous sequences, such as a helical helix motif, a spacer motif, an N-terminal oligomerization motif, a C-terminal transcription motif, an Src homology 2 motif, and a DNA-binding domain. Importantly, each STAT has a tyrosine necessary for DNA attachment, oligomerization, and nuclear transport located near the Src homology region (Heo et al., 2022). Several STATs have been linked to carcinogenesis, but STAT1, STAT3, and STAT5 are strongly intertwined with HNSCC (Yang M. et al., 2022). By altering the stabilization and functionality of the dimeric protein, *i.e.,* hypoxia-inducible factor-1α (HIF-1α), upregulation of STAT3 induces tumor angiogenesis. It also stimulates vascular endothelial growth factor (VEGF) transcription by interacting with the VEGF regulator with HIF-1α (Carbajo-Pescador et al., 2013; Elumalai et al., 2022). By triggering the expression of designated matrixins 1, 2, 9, and 10, STAT3 also facilitates cellular infiltration and proliferation (Zhu et al., 2012). HNSCC has been associated with enhanced EGFR transmission, STAT3 stimulation, and upregulation (Khatoon et al., 2022). Moreover, STAT3 was found to be an Src-based activator of EGFR-triggered development of HNSCC *in-vitro*, inhibiting the apoptotic pathway and accelerating malignant progression *in-vivo* (Qureshy et al., 2022). Moreover, regulation of STAT5 has been shown to promote HNSCC tumorigenesis, infiltration, and epithelial-to-mesenchymal transformation (Jinesh and Brohl, 2022). It was observed that STAT-5A, but not STAT-5B, is critical for the cellular uptake of HNSCC. JAK2 is not essential for the stimulation of STAT-5A, but the expression of EGFR can trigger it (Puigdevall et al., 2022). Consequently, STAT -5B, but not STAT-5A, is essential for the development of HNSCC *in-vivo* and *in-vitro*. In HNSCC, erythropoietin was also found to trigger STAT5 stimulation by JAK2 and promote cell infiltration (Wong et al., 2022). Uncontrolled JAK/STAT activation is involved in the development, dissemination, and relapse of HNSCC because it promotes multiplication, mortality, inflammation, infiltration, and angiogenesis (Mohan et al., 2022).

### 2.8 Hypoxia and angiogenesis

**FIGURE 8:**
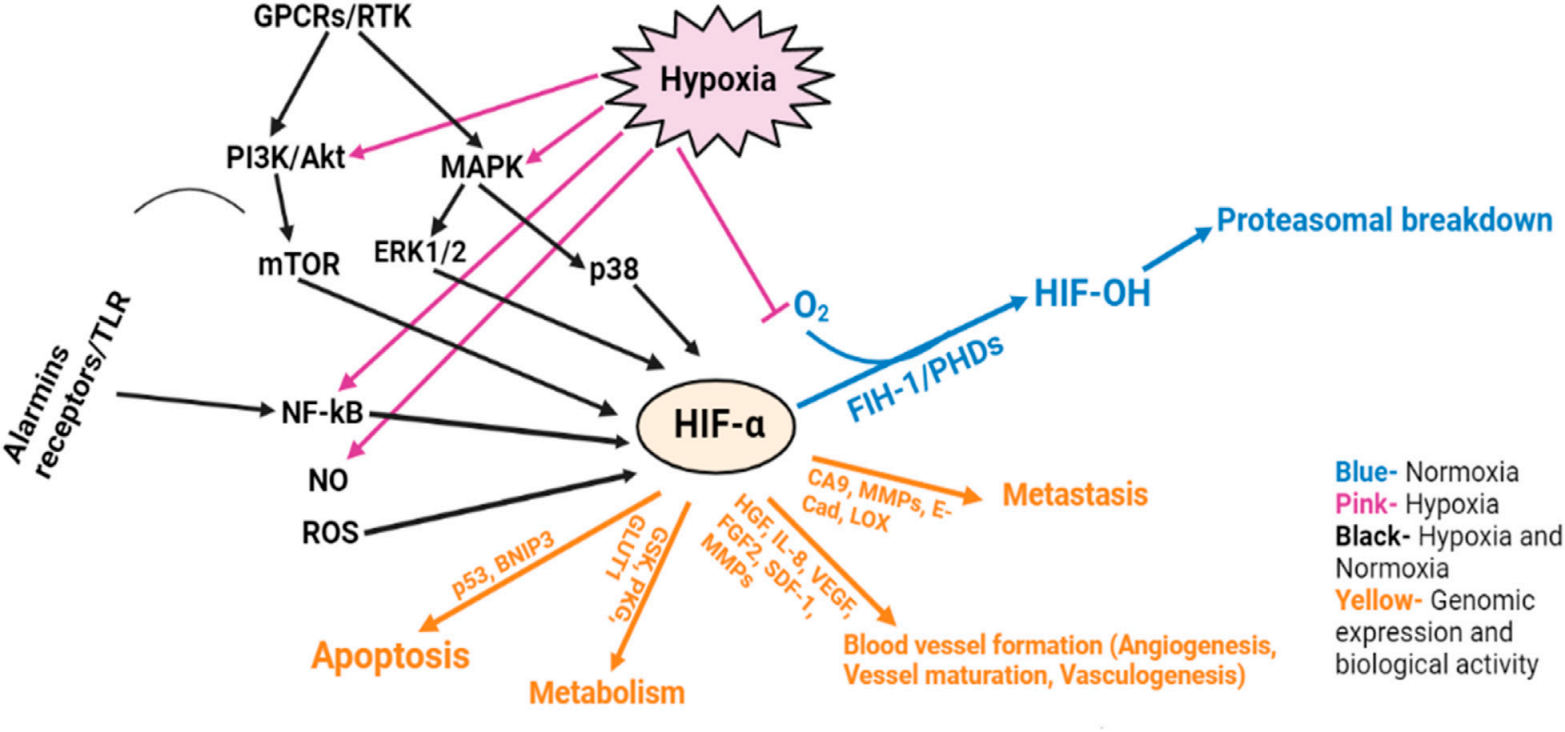
The role of hypoxia and angiogenesis in HNSCC progression.

A tissue’s cell adapt to hypoxia by initiating the finely controlled, multi-step mechanism of angiogenesis. The partial hypoxia resulting from tissues developing above the biological oxygen transport threshold causes the cells of the capillary beds, which are biologically oxygenated by the simple passage of oxygen, to release angiogenic variables, which in turn cause the capillary beds to enlarge. In the event of arterial infiltration into vascular zones, wound repair, embryogenesis, and tissue growth angiogenesis could be considered a physiological phenomenon. Tumor hypoxia occurs when oxygen availability to cancer cells is decreased or eliminated due to aberrations in microvessel morphology, impaired tissue perfusion, and significant oxygen demand related to the rapid energy metabolism of cancer cells (Zhu et al., 2021). Tumor deprivation is common in HNSCC and is related to patients’ sensitivity to therapy, poor prognosis, and reduced life expectancy (Alsahafi et al., 2019). In a hypoxic environment, the von Hippel-Lindau tumor suppressor protein ubiquitinates and eliminates HIF-1α and HIF-2α. Glucose transporter 1 (GLUT1), carbonic anhydrase 9 (CA9), VEGF, phosphoglycerate kinase (PGK), and other signaling pathways are candidates for HIF-1α, while HIF-2α drives EGFR signaling (Codony and Tavassoli, 2021). HIFs have a limited way of regulating mTOR because they respond to mTOR blockers (Cluff et al., 2022). VEGF (VEGF-A), which is in the family of homodimeric peptides, is needed for endothelial cell proliferation and the formation of new blood vessels. VEGF molecules function through the corresponding cell membrane tyrosine kinase channels, namely, VGFR1, VGFR2, and VGFR3, with VGFR2 being the major channel responsible for vascular endothelial cell proliferation, division, and motility (Miller and Sewell-Loftin, 2022). VEGF upregulation is associated with higher malignancy progression, increased sensitivity to cytotoxic treatments, poorer survival, and a more combative phenotype in HNSCC identical to hypoxia. The expression of angiogenic markers including hepatocyte growth factor (HGF), VEGF, IL -8, and fibroblast growth factor-2 (FGF2) is more pronounced in oral precancerous than in reference specimens (Figure 8). In HNSCC, two possibly independent mechanisms may regulate angiogenesis where the specimens either articulated reduced levels of FGF2 and VEGF with greater degrees of HGF and IL8, or greater degrees of FGF2 and VEGF that were linked with higher angiogenic factors. The diversity of HIF-mediated angiogenesis pathways suggests that in contrast to the fact that various molecular processes can result in the same angiogenesis phenotype. Thus, understanding the different types of angiogenic phenotypes may help in the development of more specific antiangiogenic interventions (Miller and Sewell-Loftin, 2022).

### 2.9 DNA damage repair cascade

**FIGURE 9:**
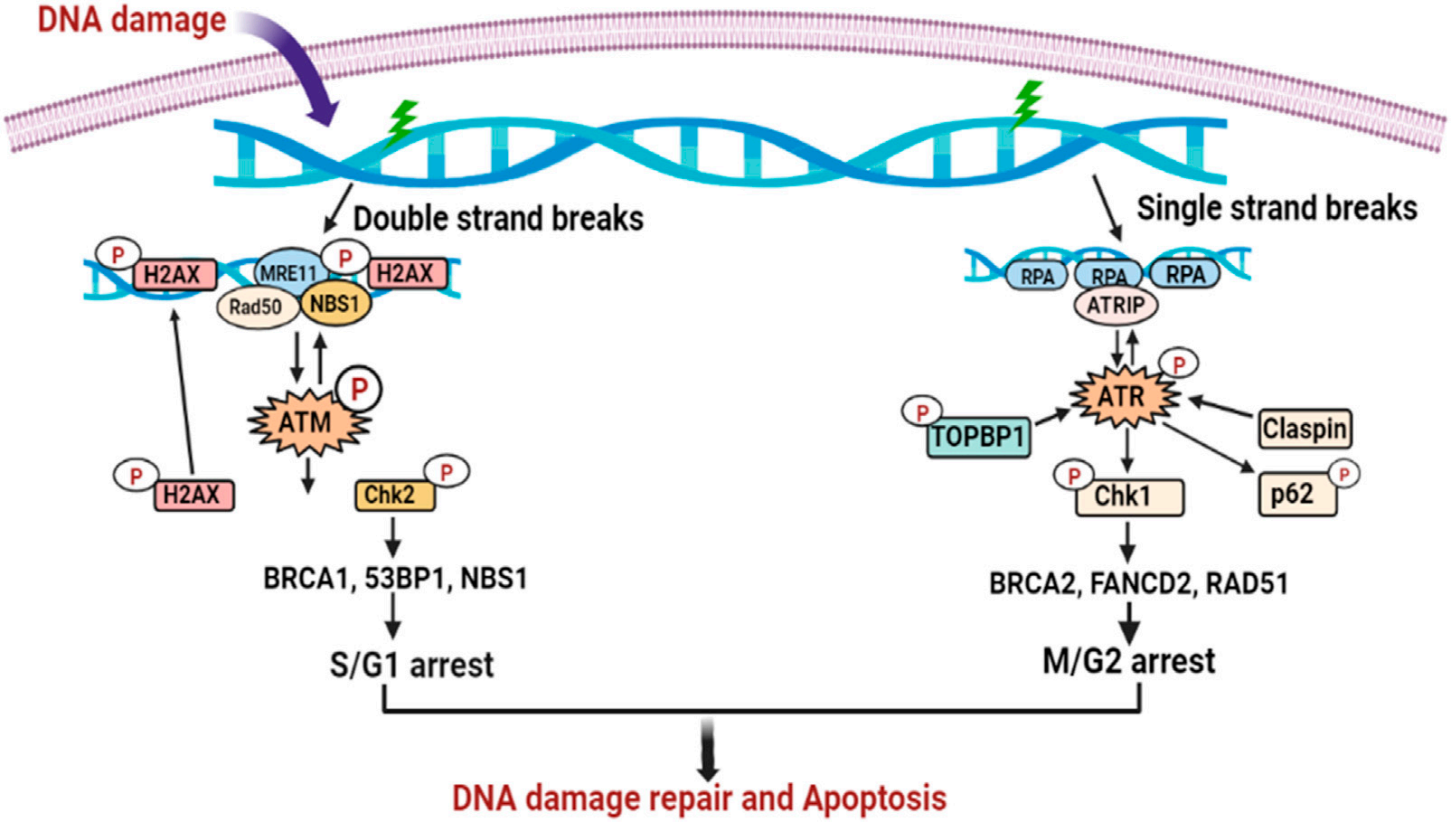
DNA damage repair cascades in HNSCC progression. The ATR and ATM DNA damage repair pathways are triggered in action to ssDNA or dsDNA breaks. Both cascades induce DNA repair by base-pairing or, alternatively, eradicate the defective cell through apoptosis.

The process by which a cell detects and maintains a disorder is called DNA repair. After DNA destruction, cell cycle regulators are triggered. To give the cell sufficient time to correct the destruction before proceeding with replication, the cell cycle is interrupted by stimulation of the regulators. The regulators of DNA destruction are widely distributed in the S/G1 and M/G2 domains. In addition to the breast cancer type 1 gene (BRCA1) and breast cancer type 2 gene (BRCA2), p53 binding protein 1 (p53BP1), a mediator of DNA damage checkpoint 1 (MDC1), and two important enzymes, namely, ATM (ataxia-telangiectasia mutated) and ATR (ataxia-telangiectasia and Rad3 related), modulate regulator signaling (Figure 9) (Xie et al., 2022). BRCA1 and BRCA2 are in the leading 30 of 236 markers that are highly frequently mutated in human papillomavirus-negative HNSCC, although other markers that are implicated in DNA destruction are also subject to genetic abnormalities in HNSCC, but at varying percentages (Chung et al., 2015). The prevalence of somatic alterations in ATM (1%–16%), ATR (4%–10%), BRCA1 (6%), and BRCA2 (7%) support the evidence that members of the DNA repair cascade are drug candidates in HNSCC (Seiwert et al., 2015).

## 3 MicroRNAs

The RNA silencing and post-transcriptional modulation of the expression of genes are mediated by small functional RNAs called microRNAs (miRNAs), which are only around 22 nucleotides short and which also involved in the various roles in the molecular signaling pathways. As of now, it has been shown that can control approximately 60% of mRNA *via* taking involvement in the multiplication, apoptosis, cell signaling, and possibly the cell’s responsiveness to stressors (Nowicka et al., 2019). At every stage of neoplasia, the aforementioned mechanisms undergo pathological alterations. This information is used to conduct miRNA analysis on every plane. The human genome contains information for over 2,000 miRNAs, albeit not all of them have been fully characterized. The initial investigation examined miRNAs in progressive lymphocytic leukemia B (PBL-B); it was evidenced that miRNAs had an impact on the function of these proteins as important promoters of both oncogenes and suppressor genes (Sayyed et al., 2021). Such a role in tumorigenesis is possible for microRNA. Certain miRNA expression is typical not only for certain organ tissues but also for certain cancers with different causes. By determining the miRNA signature for specific forms of neoplastic tumors, it is possible to effectively identify the pathological and clinical features of the changes, including proliferation, the extent of tumor heterogeneity, the potential for vasculogenesis, and the motility of cancer cells (Huang and Zhou, 2022). The stability of miRNA in the compound under study is a critical factor. This makes it simple to access the substance. miRNA has been discovered in the blood serum of solid tumors and hematological neoplasms of various origins. This might indicate an easy way to determine how far along the neoplasm process is in its development. It was also revealed that miRNA circulates in body fluids as a chemical messenger that triggers membrane receptors, suggesting that miRNA serves various functions in intracellular systems in addition to post-transcriptional control of genes. In a cancer cell, miRNAs can act as suppressor genes or oncogenes (Syeda et al., 2020). One such is miR-221, which is in a tumor suppressor gene in erythroblast leukemia as opposed to an oncogene in solid tumors (Jiang X. et al., 2019).

## 4 Role of miRNA as proto-oncogene and oncogene

The system of proper gene transcription regulation is disturbed in the altered cell. In contrast to how miRNA targets a specific oncogene in a healthy cell by suppressing it when the miRNA gene is silenced, the oncogene output experiences enhanced expression (Ahmed et al., 2022; Uzuner et al., 2022). The tumor suppressor is effectively hindered by uncontrolled amplification of the miRNA gene that modulates it, which also induces carcinogenesis. Oncogenes or tumor-suppressor genes can function as miRNAs (Ma M. et al., 2022). The initial miRNAs identified as silencers were miR-16a and miR-15o, which are expressed in site 13q14, and are present in more than 50% of those with chronic leukemia (Braga et al., 2022). Generally, miRNA gene expression is suppressed in malignant tumors, which may increase the probability of abnormal disease progression (De Palma et al., 2022). Another evidence is the distinct deletion of miR-126 expression, which promotes tumor development and propagation in bone and pulmonary malignancies, and miR-335 expression, which facilitates metastasis and serves as its hallmark in breast cancer (Galindo Torres et al., 2023).

## 5 miRNA in the detection of HNSCC

### 5.1 Tumor suppressor miRNAs in HNSCC

We retrieved information from various research indicating decreased miRNA expression in plasma and tissue samples from patients with HNSCC. In silico interpretation or structural studies in the respective papers revealed possible targets of these miRNAs. In most cases, these miRNAs modulate the expression of the gene that stimulates cellular growth or prevents apoptosis. According to Lo et al., miR-200c expression was dysregulated in the localized metastatic lymph gland of HNSCC samples, but BMI1 gene expression was overexpressed compared with the original tumors. In HNSCC cells, their structural research results showed a significant association between the 3'UTR of BMI1 and miR-200c. In extracted CD44^+^/ALDH1^+^ cells from HNSCC that exhibited cancer stem cell features (CSC), they also discovered the dysregulation of this miRNA. The oncogenic CSC -like properties of these cells could be reduced by the induced upregulation of miR-200c. Remarkably, the upregulation of miR-200c increased the expression of E-cadherin in CD44^+^/ALDH1^+^ cells but reduced the expression of N-cadherin, Snail, and ZEB1. In a mouse xenograft, the importance of miR-200c overexpression in reducing malignant behavior was also confirmed (Lo et al., 2011). Allen *et al.* investigated the effects of HNSCC patient serum on miRNA expression in challenged cells *in vitro* using next-generation sequencing (NGS) technology. Their results showed that the administration of patient serum samples induced challenged cells to produce a specific miRNA transcription pattern. These miRNAs were reported to be implicated in cancer-related mechanisms, *i.e*., apoptosis and cell cycle, as revealed by studies of signaling pathways and gene ontology (Allen et al., 2018). Table 1 shows the collection of dysregulated miRNAs and their roles in HNSCC samples.

**TABLE 1:** Summary of miRNAs dysregulated in HNSCC.

miRNA	Type of cancer	Tissues	Number of clinical specimens	Cell line	Pharmacological targets	Signaling cascades	Pharmacological action	Clinical outcome	References
miR-99a	HNSCC	Plasma and entangled tumors, and regulating tissues	16 entangled tissue specimens and 9 entangled plasma specimens from HNSCC individuals were collected before and 6 months after the tumor was successfully removed	---	SMARCA5, mTOR, IGF1R, MTMR3	---	The treatment efficacy is influenced by circulating miR-99a deregulation	---	Hou et al. (2015)
hsa-miR-29c-3p	HNSCC	Tumor biopsy specimens collected from a male patient	42 patients	---	---	---	Increased tumor stage was linked to the deregulation of hsa-miR29c-3p intumor tissue	---	Hudcova et al. (2016)
miR-128	HNSCC	---	---	JHU-22, JHU-13	Paip2, H3f3b, BAX, BAG-2, BMI-1	Apoptotic and proliferation cascades	miR-128 acts as a tumor suppressor gene	---	Hauser et al. (2015)
miR-98	HNSCC (Laryngeal, oropharyngeal, and oral)	Serum	7 healthy male volunteers and 7 male patients with HNSCC	---	---	---	Modulation of tumor metastasis	---	Martinez et al. (2015)
miR-375	HNSCC	Entangled tumors, and regulating tissues	51 patients	---	---	---	Possible prognostic value, dysregulated in HNSCC	---	Kalfert et al. (2015)
miR-32-5p	HNSCC	Serum specimens	4 healthy control and 7 patients with HNSCC	HeLa	Sirt1, MDM2	P53 cascade	Modulation of p53 in the treatment cell might be augmented by dysregulating this miRNA.	---	Allen et al. (2018)
miR-26a/b	HNSCC	Serum	7 healthy control male and 7 male HNSCC patients	---	Cyclin D2	---	Initiation of tumor-specific apoptosis, prevention of disease recurrence, and inhibition of cell growth	---	Martinez et al. (2015)
miR-376c	HNSCC	Entangled tumors and healthy specimens	40 patients	Ca9-22, Cal-27, 293T, HOKs	RUNX2	Inhibin β-A axis/RUNX2	miR-376c inhibits lymph node metastasis by Activin-A axis/RUNX2	A poor outcome for HNSCC is associated with reduced expression of miR-376c-3p	Chang et al. (2016)
miR-876-5p	HNSCC	Tumor tissues	40 patients	WSU-HN6, WSU-HN4, HEK293T, CAL27	Vimentin	---	Cell motility and infiltration are suppressed by miR-876-5p	---	Dong et al. (2018)
miR-200a, miR-93	HNSCC	Saliva specimens	Collection of 83 saliva specimens from 33 HNSCC patients that were taken repeatedly before, during, and after radiotherapy	---	CTNNB1 and ZEB2	---		---	Greither et al. (2017)
miR-205-5p, miR-124-3p, and miR-92a-3p	HNSCC	Saliva specimens	108 healthy control and 108 HNSCC patients	---	---	---	HNSCC biomarkers with high specificity and selectivity	---	Salazar-Ruales et al. (2018)
miR-29s	HNSCC	Healthy tissues and entangled tumors	23 patients	FaDU and SAS	ITGA6 and LAMC2	Focal adhesion cascade	miR-29s inhibits cancers cell motility by regulating laminin-integrin signaling	---	Ghafouri-Fard et al. (2020)
miR-150	HNSCC	Serum	7 healthy control male and 7 male HNSCC patients	---	EP300 and PIM1	---	Regulate cell division and proliferation	---	Martinez et al. (2015)
miR-203	Tongue squamous cell carcinoma (TSCC)	Surrounding non-cancerous samples and entangled tumor	10 patients	Tca8113	PIK3CA	---	miR-203 triggers the cell cycle to halt and enhances the apoptosis	---	Rastogi et al. (2017)
miR-545	Oral squamous cell carcinoma (OSCC)	Healthy tissues and entangled tumor	20 patients	KON, SAS, HSC4, HSC2	RIG-I	HPV cascade	miR-545 plays a tumor suppressor function in OSCC.	---	Yuan et al. (2019)
miR-532-3p	TSCC	Entangled tumor	23 patients	SCC-25, CAL-27, TCA8113, and TSCAA	CCR7	---	Overexpression of miR-532-3p suppresses cellular motility, and multiplication, and mediates apoptosis	---	Feng et al. (2019)
miR-204-5p	OSCC	Refrigerated samples from OSCC patients	52 patients	Human OSCC cell lines	CXCR4	NF-κB and Wnt/b-catenin signaling cascades	miR-204-5p reduced OSCC cell invasion and proliferation	---	Kalfert et al. (2015)
miR-200	OSCC	---	---	SCC15 and SCC25	EZH2	STAT3 signaling cascades	miR-200 regulates STAT3 signaling to induce anticancer actions	---	Wang et al. (2018)
miR-26a	Nasopharyngeal carcinoma	Healthy and tumor tissues	16 healthy nasopharyngeal and 18 tumor samples	HEK-293T, HONE1, 6–10B, NP69, and CNE1	CCND2 and EZH2	---	miR-26a hindered cellular proliferation partially owing to a G1-phase halt	---	Lu et al. (2011)
miR-145	OSCC	Surrounding healthy tissues and tumor tissues	48 patients	SCC-9	HOXA1	MAPK/ERK signaling cascade	miR-145 prevents cellular motility, viability, and metastasis	---	Ding et al. (2019)

### 5.2 Onco-miRNAs in HNSCC

We created a table with the information we found about miRNAs that were overexpressed in HNSCC cells compared with non-cancerous cells. In Table 2, we have compiled evidence from 63 research findings demonstrating the overexpression of miRNAs in this type of cancer. Few of these miRNAs were designated as onco-miRNAs because *in silico* modeling and functional assessments showed that they alter cancer-related signaling pathways. Ramdas et al. used the miRNA microarray method to determine how abundant miRNAs were in HNSCC and similar healthy tissues. It was found that 20 miRNAs were highly expressed in these samples. The researchers also showed that the targets of these miRNAs are dysregulated. Notable targets included TGFBR3, PDCD4, and adenomatous polyposis coli (APC), leading to the discovery that the upregulation of these miRNAs may be a factor in the dysregulation of mRNAs that regulate the development and progression of HNSCC (Ramdas et al., 2009). Kalfert *et al.* found that the levels of miR-34a, miR-200c, and miR-21 were increased in all HNSCC tumors studied. The similarity between p16 activity and miR-34a activity in malignant tumors is remarkable (Kalfert et al., 2015). Table 2 shows the collection of overexpressed onco-miRNAs and their roles in HNSCC samples.

**TABLE 2:** Summary of miRNAs upregulated in HNSCC.

miRNA	Type of cancer		Tissues	Number of clinical specimens	Cell line		Pharmacological targets		Signaling cascades		Pharmacological action		Clinical outcome			References	
miR-372	HNSCC		Adjacent non-tumor healthy tissues and entangled HNSCC	66 patients	SCC25, OECM1, FaDU, and OC3		p62		mTOR cascade		miR-372 induces HNSCC cell motility by targeting p62		---			Yeh et al. (2015)	
miR-320	HNSCC (Laryngeal, oropharyngeal, and oral)		Serum	7 healthy control male and 7 male HNSCC patients	---		PTEN and CDKN2A		---		Suppresses the cell cycle inhibitors p21 and p57 to induce multiplication		---			Martinez et al. (2015)	
miR-21	HNSCC		Healthy and tumor tissues	104 HNSCC patients	---		BTG-2, ACTA2, PDCD4		---		miR-21 might facilitate migration and proliferation by suppressing BTG-2, ACTA2, and PDCD4		---			Mireștean et al. (2022)	
miR-21, miR-34a, miR-200c	HNSCC		Healthy and tumor tissues	51 HNSCC patients	---		---		---		Feature some significant predictive value		---			Kalfert et al. (2015)	
miR-223	HNSCC		Healthy and tumor tissues	16 HNSCC patients	---		hCdc4/FBXW7		FGF cell signaling		Downregulation of miR-223 shown cancer relapse		---			Hou et al. (2015)	
hsa-miR-32-5p	OSCC		Serum and healthy and tumor tissues	5 OSCC patients	---		---		---		Biomarker for non-invasive screening of OSCC patients		---			Schneider et al. (2018)	
miR-654-5p	OSCC		Healthy tissues and entangled tumor	157 OSCC patients	CAL-27, Tca-8113		GRAP		MAPK/Ras signaling cascade		miRNA-654-5p induces invasion, motility, chemoresistance, and multiplication by modulating EMT		The miR-654 activity was associated with lymph node metastasis and a poor outcome			Lu et al. (2018)	
miR-187	OSCC		Healthy tissues and entangled tumor	19 healthy and 56 OSCC patients	293FT, OECM1, HSC3		BARX2		---		miR-187 induces metastasis and carcinogenic activity		---			Lin et al. (2016c)	
miR-5100 and miR-626	OSCC		Tissue and serum specimens	90 healthy and 218 OSCC patients	---		---		---		These miRNAs have a substantial association with OSCC outcome		Poor findings were associated with increased levels of miR-5100 and miR-626			Shi et al. (2019)	
miR-107/miR-103	HNSCC (Laryngeal, oropharyngeal, and oral)		Serum	7 healthy control male and 7 male HNSCC patients	---		NF1, KLF4, and DAPK		---		An oncomiR induces cell motility and growth		---			Martinez et al. (2015)	
miR-24	TSCC		Healthy tissues and entangled tumor	79 TSCC patients	8 TSCC cell lines		PTEN		Akt/PTEN cascade		Specifically inhibiting PTEN, miR-24 promotes cell viability, migration, and tolerance to cisplatin		---			Zheng et al. (2015)	
miR-450a	OSCC		Healthy tissues and entangled tumor	35 OSCC patients	SAS and DOK cells		TNF-α/TMEM182		NF-B and ERK cascades		In OSCC, miR-450a regulates cellular survival and metastasis		Elevated OSCC cellular invasion potential induced by high expression of miR-450a			Hsing et al. (2019)	
miR-122-5p	HNSS		Saliva specimens	108 healthy and 108 HNSCC patients	---		---		---		A unique biomarker for the identification of HNSC		---			Salazar-Ruales et al. (2018)	
hsa-miR-375	OSCC		Serum and healthy and tumor tissues	5 ONSCC patients	---		MMP13		---		Enhances virulence and metastatic potential		---			Schneider et al. (2018)	

### 5.3 miRNA as prognostic marker

Although cancers of the same type generally show considerable genetic variation, this is often neglected in therapeutic interventions and may lead to the choice of inappropriate and thus inefficient treatment. The potential for accurate and, more importantly, highly specific detection of the cancer type enables the ideal personalized treatment choice for the patient and greatly increases the likelihood of survival (Antra et al., 2022; Han et al., 2022). In HNSCC, a point mutation in the leader sequence of Kirsten rat sarcoma 3 (KRAS), originating in the complementary region of the miRNA let-7, has been associated with disease development and patient longevity. This association was associated with significantly reduced patient longevity, raising the possibility that this mutant version could alter the genetic composition of the disease or the response to treatment (Ulusan et al., 2022). When the TP53 gene is mutated, the tumor suppressor protein p53, which is one of the most frequently dysregulated proteins in neoplasms, is examined for its longevity chances in patients with HNSCC. This study revealed a reduced overall longevity with an even stronger association with obstructive genetic variations (Nathan et al., 2022). These results were validated by other p53 studies, which also improved the information by indicating that this association was stronger in the diagnostic category of patients treated prophylactically after resection. miR-375 has shown promise as a predictive biomarker of poor prognosis and tumor growth in HNSCC and may act by inhibiting the intrusive properties of the tumor. This is relevant to both metastatic development and reduced life expectancy (Jayaseelan and Arumugam, 2022). Increased expressions of hsa-miR-210 have been associated with the occurrence of distant metastases and poor prognosis, indicating that miRNA can also be used to predict the likelihood of relapse (Turai et al., 2022). A study by Thomaidou et al. showed that decreased expression of hsa-miR205 is strongly associated with recurrence of distant metastases, independent of the extent of disease at the time of detection and therapy. Moreover, the prognosis of HNSCC is positively correlated with aggregate decreased activity of hsa-let-7d and hsa-miR-205 in HNSCC tumors (Thomaidou et al., 2022). The findings on how high mobility group protein 2 (HMGA2) modulates mutagenic responses are important to consider, as this information could influence the effects of chemotherapeutic agents. In HNSCC, HMGA2 is associated with enhanced specific chemosensitivity to the topoisomerase inhibitor II (doxorubicin) (De Martino et al., 2022). HPV-16-induced malignancies are a separate category of HNC, they are more commonly observed in the oropharynx, and since HPV-infected epithelial cells are very sensitive to chemotherapy, this category has a better chance of cure (Zhou et al., 2022). hsa-miR-139-3p can be dysregulated by HPV-16, which can promote the development of cervical cancer and HNC, and researchers believe that this is due to the viral alteration of host miRNA transcription (Sannigrahi et al., 2017). Since the tests were performed with salivary miRNAs, it is also possible to distinguish between HPV-positive and HPV-negative HNSCCs based on the miRNA profile. The researchers also claimed that salivary miRNA sequences can distinguish between different HNSCC tumor stages (Salazar-Ruales et al., 2018). Several miRNAs, including miR-195-5p, miR-142-3p, miR-374b-5p, miR-574-3p, and miR-186-5p, have been reported as an HPV-independent prognostic profile for HNSCC patients who received combinatorial radiochemotherapy (Galindo Torres et al., 2023).

### 5.4 Circulating miRNAs as a fluid-phase biopsy

In recent years, liquid phase biopsy has become increasingly important for the rapid detection of malignant disease, especially HNSCC. It is a rapid and simple assay that looks for tumor-derived extracellular vesicles (EVs), circulating tumor cells (CTCs), and circulating tumor DNA (ctDNA), which are discharged into the peripheral circulation from malignant tumors and their metastases regions (Mattox et al., 2019). Regardless of how interesting this approach appears, it must be recalled that it is novel and currently requires a lot of studies, and because it is still in its initial phases, it addresses the issue of an absence of uniform protocols and consistency (Provenzano and Allayeh, 2020). The US Food and Drug Administration (FDA) certified the liquid biopsy examination for the first time in 2017 as a consequence of the advantages and possibilities of this kind of analysis and the tremendous pace at which innovation is progressing in this direction (Kwapisz, 2017). The limited investigations that have been reported so far on the application of miRNA as a screening tool in liquid biopsy for HNSCC look intriguing. Mazumder *et al.* highlighted the significant functionality of miR-371, miR-338, miR-146a, and miR-134 as metastasis variables as well as miR-7d, miR-21, miR-150, and miR-371 as prognostic variables in oral carcinoma (Mazumder et al., 2019). Furthermore, the prognostic variables miR-21 and miR-7d were observed to be substantially associated with treatment resistance. The potential use of miRNA in fluid-phase biopsy for the detection of oral squamous cell carcinoma (OSCC) was exhaustively examined in the 2019 systematic review by Rapado-González *et al* (Rapado-González et al., 2019). The researcher states that conventional biopsy is currently the golden seal of approval and that this fact is mainly due to the low confirmation of miRNA variables and the extremely high level of tumorigenesis. Tumor heterogeneity continues to be a major primary challenge in both detection and therapy (Ramón y Cajal et al., 2020). An additional problem in the effort to exploit miRNA as a marker is the extremely significant incidence of heterogeneity linked to HNSCC. Given the heterogeneity of malignancy, an optimal marker must detect a specific form of cancer, while a significant degree of sensitivity introduces the possibility that not all incidences will be detected (Canning et al., 2019).

### 5.5 Radiotherapy and immunotherapy markers

Radiotherapy (RT) is an effective therapeutic strategy for people suffering from HNC. Although progress in therapy, many cancers become resistant to radiotherapy, which lowers mortality chances. In HPV-positive HNSCC, aberrant intracellular DNA damage sensitivity processes, particularly DNA double-strand fragment signaling and restoration, are known to be major contributors to variable radiosensitivity (Fabbrizi and Parsons, 2020; Hutchinson et al., 2020). Considering each patient’s biological response to RT is unique, there is a significant need for biomarkers that can be used to assess treatment efficacy, categorize patients appropriately, and provide tailored treatment. Consequently, although most patients with a localized progressed disease can be treatable with a cocktail of chemotherapy, surgery, and/or radiotherapy and survive, others will experience recurrence or distant metastasis and are regarded as untreatable (Bauml et al., 2019). Immunotherapy may be the best course of treatment for these patients. The potential for responses to be highly enduring, with prognostic value often assessed in years, makes immunotherapy a significant advantage over conventional types of systemic chemotherapeutics. The search for novel biomarkers will be important for optimizing the efficacy of immunotherapy in patients with advanced HNSCC, as most lack a defined tumor-specific target (Cortez et al., 2019). Table 3 lists the miRNAs that have been postulated as markers in radiation and immunotherapy.

**TABLE 3:** miRNA as diagnostics markers for radiation and immunotherapy.

MiRNA	Regulation	Therapeutic utility	References
**Radiotherapy**			
miR-301a, miR-18b, miR-141	Downregulation	Tolerance to RT marker	Chen et al. (2022a)
miR-1323, miR-34c-5p, miR-371a-5p	Upregulation	Tolerance to RT marker	Chen et al. (2022b)
miR-200a, miR-93	Upregulation	Therapy assessment following radiation	Chen et al. (2022a)
miR-29b, miR-16, miR-1254, miR-150	Upregulation	Tolerance to RT marker	Hutchinson et al. (2020)
miR-296-5p	Upregulation	Tolerance to RT marker	Maia et al. (2015)
**Immunotherapy**			
Let-7 family	Downregulation	Immunotherapy predictive marker	Yu et al. (2019)
miR-28-5p, miR-21-5p, miR-199a-3p	Downregulation	Immunotherapy predictive marker	Li et al. (2018)
miR-28-5p, miR-21-5p, miR-200c-3p	Downregulation	Marker of the anti-PD-1/PD-L1 therapy responsiveness	Shukuya et al. (2020)

## 6 Nanomedicine (NM) for HNSCC therapy

A number of nanocarriers can be used for the diagnosis and treatment of HNSCC. These include lipid-based nanocarriers, metallic nanoparticles, dendrimers, polymeric nanoparticles, and carbon nanostructures. Because they are so small and have unique biological and physicochemical properties, they have great potential as transporters for bioactive compounds (Swain et al., 2016; Hinge et al., 2020; Sahu et al., 2021; Setia et al., 2022). There are two categories of nanoparticles: inorganic and organic particles, based on their chemical composition. They are mostly used as magnetothermal probes and photothermal drugs, as carriers of gene vectors, and as radiation enhancers for the treatment and detection of cancer. Noble metals, quantum dots (QDs), lanthanide-based nanoparticles, and metal oxides are some examples of the inorganic components that make up hard nanoparticles. Because these nanoparticles are composed of inorganic components, they can be toxic and are difficult to hold together in some serious situations. Soft nanoparticles, on the other hand, are composed of organic components and include liposomes, lipid carriers, polymeric nanoparticles, and dendrimers. The surfaces of soft nanoparticles can be modified with a wide range of amphiphilic and hydrophilic compounds, including fluorophores or substances that normally confer stability to poorly soluble molecules *in vivo* or reduce their ability to be recognized by macrophages (Andrade et al., 2016; Caparica et al., 2020; Wojtynek and Mohs, 2020; Kaurav et al., 2023). Nanoparticles can have adverse effects depending on their shape, hydrodynamic diameter, systemic half-life, chemical groups on their surface, and route of administration. Larger nanoparticles are very reactive and more hazardous than small nanoparticles due to their larger contact area (Khan et al., 2022). Table 4 provides an overview of the different applications of nanocarriers in the treatment of HNSCC. These applications are discussed in the following sections.

**TABLE 4:** Summary of a variety of applications of nanomedicines in HNSCC regulation.

Nanocarrier	Fabricated	Targettable site	Targeted tissue/Cell	Method of preparation	Advantage	Experimental outcomes	References
Gadolinium-based nanoparticles (AGuIX^®^)	1,4,7,10-tetra-aza-cyclo-dodecane-1-glutaric anhydride-4,7,10 tri-acetic acid (DOTAGA)	---	HNSCC cells lines (SQ20B)	---	Enhanced the effectiveness of the anti-cancer drug, and radiation therapy, and minimize toxicity	Procedure for radiotherapy treatment. Following cellular ingestion and subsequent aggregation in lysosomes, a novel AGuIX^®^ preparation has the ability to radiosensitize HNSCC. While radiation itself induced mitochondrial dysfunction and delayed cell death, pre-treatment by (AGuIX^®^) resulted in extensive DNA fragmentation	Simonet et al. (2020)
Gold Nanorods (AuNRs)	Rose Bengal (RB)	---	OSCC	Seed-mediated growth	Greater efficiency, better physical stability, and small size	Photothermal therapy (PTT) and photodynamic treatment (PDT) for oral carcinoma. The ingestion of RB by cancerous cells is augmented by the RB-AuNRs, which also demonstrate better photodynamic effectiveness. The combination of PTT-PDT characteristics of RB-AuNRs has the potential to treat cancer and offer superior restorative benefits on oral carcinoma than either PTT or PDT used singly	Wang et al. (2014)
Gold nanoparticles (NPs)	Anti-EGFR monoclonal antibody	EGFR	OSCC	Reduction process	Reduced toxicity, excellent stability, and promising bio-distribution	Tailored drug administration of the photothermal agent and anti-EGFR antibody	Knights et al. (2020)
Biocompatible polymer [Poly (lactide-caprolactone): Poly (e-caprolactone)]	Cisplatin and CCL21 (Chemokine ligand 21)	---	HNSCC tumors	Nanoprecipitation	Enhanced stability and accumulation of drugs	Administration of Cisplatin and CCL21. Effective anticancer responses and a reduction in tumor size are evidenced by CCL21, cisplatin, and the CCL21-cisplatin combination polymer. Additionally, using cisplatin polymer in conjunction with radiation treatment potentially allows patients who have already administered the drug to undergo less radiotherapy. Histopathology revealed no inflammation or evidence of severe cytotoxicity following cisplatin-based polymer therapy and radiation	Pellionisz et al. (2018)
Cationic lipid NPs	pre-miRNA-107	miRNA-107	HNSCC cell lines (CAL27, SCC25, and SCC15 cells) of mouse xenograft model	High-pressure homogenization	Improved bioavailability, permeability, solubility, and biodistribution	Tailored pre-miRNA-107 administration. Throughout the experiment, authors explored the effectiveness and efficiency of cationic lipid NPs in delivering pre-miRNA-107 (LNP-pre-miRNA-107) targeting HNSCC tumors both *in-vitro* and *in-vivo*. miRNA-107 distribution into HNSCC cells was more than 80,000 times higher with LNP/pre-miRNA-107 than with normal pre-miRNA-107. After LNP/pre-miRNA-107 administration, the expression of HIF1-β, CDK6, and PKC was reduced compared to normal pre-miRNA-107	Zarrintaj et al. (2019)
Magnetic iron oxide NPs	---	---	Xenograft mouse model of HNSCC cell line (Tu212)	Sonochemical	Enhanced biodegradability, biocompatibility, and solubility	*In-vivo* investigational outcomes, during the initial 5–10 min, the tumor core temperature increased substantially from close to atmospheric temperature to approximately 40 °C. Based on the histopathological findings, the extensive ulcers of the treatment tumor surfaces that were not observed in the untreated group were caused by hyperthermia-induced apoptosis	Farzanegan and Tahmasbi (2022)
Polymer micelles-Poly(ethylene glycol)-poly(glutamic acid) block copolymers	Cisplatin	---	Oral HNSCC cell lines (HSC-4, HSC-3, OSC-20, and OSC-19)	Solvent evaporation	Improved accumulation and retention of the drug encapsulated and prolonged circulation time	In this work, researchers investigate the effectiveness and tolerability of polymeric micelles encapsulating cisplatin in the treatment of OSCC. Different OSCC cell lines were examined *in-vitro* for tumor inhibition potential. In mice, researchers investigated polymeric micelles encapsulating cisplatin *in-vivo* anticancer effectiveness. Polymeric micelles encapsulating cisplatin formulation showed the highest reduction in tumor size as compared to free cisplatin	Su et al. (2019)
Gadolinium NPs	---	---	HNSCC cells lines (Cal33, FaDu, and SQ20B)	---	Enhanced the effectiveness of the anti-cancer drug, and radiation therapy, and minimize toxicity	Improvement of radiotherapy. Integrating Gadolinium NPs + radiation dramatically slowed tumor development, increasing delayed apoptosis and reducing cellular growth	Simonet et al. (2020)
Superparamagnetic iron oxide NPs (SPIONPs)	Mouse anti-human cancer stem cell marker (CD44) antibody	CD44	HNSCC cancer stem cells (CSCs)	Coprecipitation	Enhanced biodegradability, biocompatibility, and solubility	CD44-SPIONPs had negligible adverse effects affecting CSCs, demonstrating their excellent biocompatibility. Tailored SPIONPs and an alternating magnetic field can inhibit CSCs, and magnetic fluid hyperthermia can greatly slow the development of transplanted Cal-27 tumors in murine	Su et al. (2019)
Dextran-coated superparamagnetic iron oxide NPs functionalized with hyaluronic acid (HA-DESPIONs)	---	CD44	TSCC	Coprecipitation	Reduced toxicity, improved biocompatibility, biodegradability, and stability	Cell death was exclusively accelerated in the CD44+ ve group subjected to HA-DESPIONs as compared to normal DESPIONs when the magnetism was triggered, based on the dual labeling of apoptosis and CD44. The initial apoptosis percentage in CD44+ ve cells improved from 1.4% to 27.5%	Thompson et al. (2017)
PEGylated nanostructured lipid carriers (NLCs)	Paclitaxel and cisplatin	Folate receptor	FaDu cells	Microemulsion	Enhanced risk/benefit ratio and excellent physical stability	Tailored co-administration of paclitaxel and cisplatin. In this experiment, Paclitaxel and cisplatin were encapsulated in NLCs. The drug-encapsulated NLCs (FA-Paclitaxel-Cisplatin-NLCs) were adorned with a synthetic form of folate-PEG-DSPE. *In-vitro* assays on FaDu cells revealed that FA-Paclitaxel-Cisplatin-NLCs had the maximum cytotoxicity and combinatorial efficacy of the dual drugs. In a mouse FaDu cells model, the *in-vivo* investigation showed the formulation with the highest tumor inhibition efficacy compared to Paclitaxel-Cisplatin-NLCs and single-drug treatment	Yang et al. (2017)
Phospholipid complex NPs	Salvianolic acid B (SalB)	---	HNSCC cells lines (HN30 and HN13)	Solvent evaporation	Enhanced retention time, greater efficiency, and reduced toxicity	Investigations on cellular ingestion, particularly quantitative and qualitative revealed that SalB cytoplasmic abundance was much greater after HN30 and HN13 were treated with SalB-PLC NPs as compared to normal SalB. Cell death activation and cell cycle inhibition were equally reported in the HNSCC cells that had been challenged to SalB-PLC NPs	Li et al. (2016)
Gold nanorods (AuNRs)	---	---	HNSCC tumor	Seed-mediated growth	Greater efficiency, better physical stability, and small size	Electropermeabilization was exploited to initially fabricate AuNRs inside platelets, and the resulting AuNRs-fabricated platelets (AuNRs-Platelets) maintained the prolonged blood flow, cancer-targeting properties of platelets, and excellent photothermal characteristics of AuNRs. Researchers establish that the treatment of AuNRs-Platelets and integration laser therapy might successfully restrict the progression of HNSCC utilizing a gene-knockout mice paradigm	Rao et al. (2018)

### 6.1 Lipid-based nanocarriers

Phospholipids make up the majority of lipid-based nanocarriers. Due to their unique properties, they arrange into ordered nanostructures in an aqueous medium. A study of encapsulated curcumin-difluorinated (CDF) in liposomes investigated proliferation suppression in cisplatin-resistant HNSCC cell lines and reported that after treatment with CDF-encapsulated liposomes, the inhibition of growth *in vitro* and the production of cytokines, growth factors, and cancer stem cell markers (CD44) were examined. The results showed that growth was greatly slowed down and CD44 production decreased, proving that liposomal CDF has a suppressive effect on carcinoma stem cells (Basak et al., 2015).

Another study was performed by using another method to transport and improve the bio-distribution of natural products studied for the chemoprevention of HNSCC. They proposed the encapsulation of salvianolic acid B (SalB) in nanoparticles loaded with phospholipid complexes (PLC-NPs) as a potential therapy for HNSCC cells (HN13, HN30) and Leuk1 precancerous cells. The results showed that intracellular aggregation of SalB was greater when HN13, HN30, and Leuk1 cells were exposed to the SalB-PLC-NPs complex (nano-SalB) than when free SalB was delivered to the cells. Like free SalB, these nanoparticles decreased cell viability and increased apoptosis (Basak et al., 2015).

For the combined administration of cisplatin (DDP) and paclitaxel (PTX) to HNC cells (FaDu cells), PEGylated nanostructured lipid carriers (NLCs) complexed with folate (FA) (FA-DDP/PTX NLCs). The folate receptor (FR) is abundant in cancer cells, especially HNC cells. Therefore, the FA-DDP/PTX NLCs enhanced the antitumor effect of the drugs while preventing apparent damage *in-vivo*. These results demonstrate the potential of this nanomaterial as a mediator for the simultaneous delivery of DDP/PTX (Yang et al., 2017).

Recent research used SLNs as a delivery system for drugs with anticancer activity in HNC and precancerous cells, including precancerous leukoplakia (Leuk1), human immortalized oral epithelial cells (HIOEC), HN30, and HN6, and showed that the 50% inhibitory concentrations (IC50) across Leuk1, HIOEC, HN30, and HN6 cells were significantly lower in the presence of included andrographolide (ADG-SLN) compared with free andrographolide (ADG). In addition, the results implies that relative to free ADG, ADG-SLN has efficient suppressive efficacy towards head and neck cancer and premalignant tissues, also showed that cell cycle arrest and cell killing worked better, suggesting that the use of SLNs as nanocarriers leads to advances in therapy (Li H. et al., 2022).

### 6.2 Polymer-based nanocarriers

Polymer-based nanostructures are stable, biocompatible, non-toxic, have few side effects, and are completely degraded in the body (Chandrakala et al., 2022). Polymer nanoparticles can be made from either synthetic or natural polymers depending on their chemical constituents. One of the factors that make cancer grow is the proto-oncogenic tyrosine-protein kinase Src, a non-receptor tyrosine kinase and one of the main targets in HNSCC. It is upregulated and hyperactivated in this type of malignancy (Shen et al., 2020). In light of this, Lang et al. developed multipurpose polymer nanoparticles (Lineardendritic mPEG-BMA4) to investigate the anti-tumor efficacy and feasibility of saracatinib-loaded nanoparticles *in-vivo*. The saracatinib-loaded polymeric nanoparticles were more effective than the drug alone in combating cancer and stopped metastasis (Lang et al., 2018). According to a reported study, cisplatin was transported with an injectable biopolymer to treat patients with HNSCC. Compared with free cisplatin, this biodegradable polymer (Atrigel^®^ leuprolide acetate) released 80% of its cisplatin over the course of 7 days and had a much stronger tumor suppressive effect being applied at each maximum tolerated dose, as opposed to unbound cisplatin. (Chen et al., 2003). Additionally, another research also applied this strategy to oral squamous cell carcinoma (OSCC) cells. They prepared polymeric micelles containing cisplatin (NC-6004) in poly(ethylene glycol)-poly(glutamic acid) block copolymers (PEG -P[Gu]) and investigated their efficiency and reliability under both *in-vitro* and *in-vivo* conditions. Finally, NC-6004 showed comparable anticancer activity to the free drug *in-vivo*, while it had a much lower growth inhibitory effect *in-vitro*. As for adverse effects, animals infused with NC-6004 were essentially free of renal cell toxicity, while renal cell apoptosis occurred in animals receiving the free drug (Endo et al., 2013). Another way was found to deliver PTX in polymeric nanocarriers to treat squamous cell carcinoma of the hypopharynx (FaDu) in animal models. The structure of the nanocarrier was developed by combining a methacrylate variant of alpha-tocopheryl succinate (α-TOS), which serves as a hydrophobic domain, with copolymers of polyethylene glycol (PEG), which have a hydrophilic domain. The study found that PTX when administered in this nanocarrier, was much more effective against cancer than when administered alone and caused apoptosis. It also proved to be safer. The fact that - TOS can produce significant amounts of reactive oxygen species (ROS) that initiate the apoptotic cascade may have influenced these results. Since it enhances the effect of PTX, the use of α- TOS in a nanoparticle formulation and the straight application of PTX-loaded polymeric nanoparticles based on α- TOS at the site of tumor growth is therefore a viable method for the treatment of HNC (Riestra-Ayora et al., 2021).

### 6.3 Metallic-based nanoparticles

Metal-based nanoparticles are also used as drug-delivering nanocarriers in the treatment of HNC. Superparamagnetic nanoparticles were one approach that involved surface functionalization. The recent research was conducted by developing a novel drug delivery method for HNC based on magnetic nanoparticles. This system consisted of a mixture of superparamagnetic, biocompatible, mesoporous Fe_3_O_4_ nanoparticles attached to polyacrylic acid (PAA). The drug used to treat HNC was bleomycin (BLM), which is either entrapped in the mesoporous shell of the superparamagnetic nanoparticles or linked to the surface of the PAA polymer by molecular cross-linkers. This polymer shell reduces the intrinsic removal of the superparamagnetic nanoparticles (MNPs) while controlling the delivery of the drug. These paramagnetic nanoparticles transported BLM to the focal point of tumor tissue, enabling its progressive release and tumor cell death while minimizing the negative effects of BLM on healthy cells and tissues. This novel approach, based on a simple methodology rather than complex technologies, was able to target the drug *in-vitro*. When cells treated with BLM-MNPs were compared with BLM-only cells and cells treated with BLM-MNPs with MNPs or cells treated with normal saline (NS) only, it was found that the apoptotic fractions were significantly higher in the cells treated with BLM-MNPs. These findings clearly demonstrate that BLM-MNPs induce apoptosis in CNE2 and Cal-27 cells and prevent tumor progression *in vivo*.Analytical investigation revealed that after therapies with MNPs and NS, BLM-MNPs and BLM, sequentially, tumor masses *in vivo* expanded with the feeding interval. Administration of BLM-MNPs and BLM stopped the expansion of tumor masses compared to NS and different control subjects with restorative efficiency and fewer side effects, offering great potential for the use of nanomedicine in the treatment of HNC (Zhang Z. et al., 2020).

### 6.4 Hyperthermia

When an area of the body or the entire body is exposed to temperatures that are higher than the normal physiological (40°C–45°C), this condition is known as hyperthermia (HT) (Zhao et al., 2013). Based on where it is utilized, the National Cancer Institute defines three forms of hyperthermia: local hyperthermia, regional hyperthermia, and total body hyperthermia (Dias et al., 2022). Along with surgery, radiation, chemotherapy, and immunotherapy, hyperthermia is being applied to treat cancer (Gao et al., 2016). This therapeutic approach enhances clinical outcomes and lessens the toxic effects of standard therapies. From a therapeutic perspective, it is not possible for it to be used as a therapeutic technique and must be paired with radiation and/or chemotherapy (Gayol et al., 2023). Nanotechnology advancements have made it possible to create novel hyperthermic agents, such as nanoparticles, which can intensify the impacts of hyperthermia by absorbing heat from outside sources. The principal source of heat is provided by nanoparticles, which also alter the route of heat loss. In order to minimize potential impacts on nearby tissues, such nanoparticles can induce heat degradation in a confined way to the targeted area (Zhao et al., 2013). Due to their superficial anatomic locations, HNC cells respond well to localized hyperthermia. The majority of the investigations used local HT technology, which in many cases only offered confined thermal dose control, and for a period of time, the devices could only be used to manage target areas within the skin. However, with the advancement of photothermal therapy (PTT), HYPERcollar3D, and nanomaterials confined HT will be able to transcend the limitations of stance, temperature, and dosing frequency control in therapeutic applications (Gao et al., 2016).

### 6.5 Plasmonic photothermal

Silica-gold nanoshells (SiAuNS) were prepared by Trinidad et al. They consist of a dielectric core (silicon dioxide) that can trap light at near-infrared (NIR) frequencies for plasmonic photothermal therapy (PPTT). Since macrophages (Ma) are attracted to hypoxic and necrotic areas in tumors, the researchers used SiAuNS-loaded macrophages (Ma) to deliver the nanoparticles in their study. They examined both the individual and combined effects of photodynamic treatment (PDT) and SiAuNS-loaded Ma-mediated PPTT on HNSCC cells. In both therapies, the tumor area is irradiated with NIR light that triggers photoresponsive molecules (in this case, AlPcS2a), resulting in the formation of ROS (PDT) or an increase in temperature (PPTT). When irradiated (5 min) with a heat flux density of 14 or 28 W/cm^2^, the durability of SiAuNS-loaded Ma decreased to 35% and 12% of the reference values, respectively, whereas no detectable cytotoxicity was observed for the non-loaded Ma under the same PPTT conditions. Thus, the absorption of SiAuNs by macrophages was confirmed. FaDu and SiAuNS-loaded Ma were mixed in 2 different ratios, 1:1 and 2:1, to test the efficacy of these interventions in HNSCC (FaDu: MaNS) at a ratio of 1:1 and an irradiance of 14 W/cm^2^, 50% of the cells survived, in contrast to only 35% of the AuNS-loaded PPTT-treated cells. The PTT effects on AuNS-loaded Ma and fused cells did not change at an irradiance of 28 W/cm^2^ (Trinidad et al., 2014). According to biodistribution experiments, AuNR-loaded PLTs showed systematic flux after 48 h, indicating that they can evade detection. Due to the affinity of PLTs for wounded regions, it was also found that PPTT medication enhanced the focusing of AuNR-loaded PLTs on the tumor, demonstrating the special self-enhancing property of PLT-PPTT in cancer therapy. Both local laser irradiation and administration of AuNR-loaded PLTs successfully slowed the development of HNSCC cells (Trinidad et al., 2014).

### 6.6 Photo-thermal probe

Photothermal and magnetic thermal probes are useful for the destruction of cancer cells (Alalaiwe, 2021). The use of a photosensitizer, such as gold, is the basis of the principle of photo-thermal radiation. Once the photosensitizer is activated by a certain wavelength range of light, it releases vibrational energy in the form of heat to terminate the cell it has absorbed. Under an alternating magnetic field, the magnetic particle has the ability to produce heat through hysteresis loss (Kwizera et al., 2022). Pavuluri et al. demonstrated the validity of this idea by selectively destroying cancer cells using submicron magnetic particles under the influence of a pulsed or high-frequency electromagnetic field that is external and has no effect on healthy cells (Pavuluri and Sheth, 2022). Depending on the temperature utilized, several processes are used to destroy cells: hyperthermia (42°C–46°C) might cause apoptosis, whereas thermal ablation (>46°C) causes necrosis (Aldaoud et al., 2022). These probes can be used for imaging in addition to cell ablation. Therapeutic use of magnetic resonance imaging (MRI) requires a contrast agent to differentiate between normal and pathologic tissue, and a number of iron oxide dishes with gold nanoparticles have shown good transverse relativity (Das P. et al., 2019). While these probes have demonstrated efficacy *in vitro*, their application *in vivo* presents a number of problems. The biggest obstacle is improving tumor-specific bio-distribution. The best method to improve tumor-specific bio-distribution is antibody conjugation. The epidermal growth factor receptor (EGFR), which is highly expressed in a variety of tumor types, has recently emerged as an ideal target for tumor identification. In contrast to non-targeted gold nanoparticles, anti-EGFR-conjugated gold nanoparticles (30 nm) intravenous injection in 2011 was found to increase the identification of HNSCC transplanted in naked mice by CT imaging (Khademi et al., 2019).

### 6.7 Magnetic probe

The scientific community has shown great interest in the application of magnetic nanoparticles of biomaterials for the targeted thermal treatment of cancer. The use of magnetic particles to increase the temperature of various tissue types when exposed to a magnetic field was the first case of magnetic substances being used as hyperthermia activators in 1957 (Ahmadi Kamalabadi et al., 2022). Concurrently, a number of papers have reported various magnetic materials, such as different kinds of nanoparticles along with radiation or chemotherapy, to evaluate the effects of hyperthermia (Venugopal et al., 2016). Each superparamagnetic nanoparticle consists of a magnetic core and a polymeric shell containing various medical and targeting components. Cancer stem cells (CSCs) play a crucial role in the growth, metastasis, and therapeutic sensitivity of HNSCC tumors. One such CSC marker is the cell-surface glycoprotein CD44. Dextran-coated superparamagnetic iron oxide nanoparticles functionalized was used with hyaluronic acid (HA)-HA-DESPIONs to investigate novel strategies for targeting the CD44 group for both therapy and visualization. When exposed to an AMF, tongue squamous cell carcinoma cells (UT-SCC-14) were employed to assess the hyperthermic potential of HA-DESPIONs or non-HA-coated DESPIONs. Cell mortality was only elevated when the magnetic field was active and in the CD44-positive group that was subjected to HA-DESPIONs, according to double labeling for CD44 and apoptosis. The upregulation surface receptor CD44 allowed HA-DESPIONs to successfully aim UT-SCC-14 cells as the apoptotic rate rose from 1.4% to 27.5% in the CD44-positive cells. A considerable regional temperature rise caused by the stimulation of HA-DESPIONs brought about by the existence of an AMF finally caused the apoptosis of tumor cells (Venugopal et al., 2016). In a similar research, a mouse xenograft model of an HNSCC cell line was used to investigate the potential of magnetic iron oxide nanoparticles-induced hyperthermia for treating HNC (Tu212). Histology findings showed that the borders of the treated tumors had ulceration (but not in non-treated controls). Furthermore, the finding of epithelial necrosis in the tumor wall of one treated tumor suggests that necrosis is the cause of hyperthermia-mediated cell death. These findings point to prospective approaches for inhibiting tumor development, treatment resistance, and metastasis, through the use of tailored magnetic nanoparticles (Zhao et al., 2012).

### 6.8 Theranostics

Nanoparticles are utilized in theragnostic techniques, which unite diagnostic and treatment in a unified system, in addition to medicinal and screening applications. Theranostic technologies, including liposomes, gold, and iron-based nanomaterials, and several others, can enable active and passive targeted, trigger the release of drugs, and carry out various therapeutic tasks while being less intrusive than traditional diagnosing methods (Huang and Lovell, 2017). Quadrapeutics are one type of multifunctional nanocarrier used in HNC treatment that can be used for the identification and elimination of HNSCC. The four therapeutic modalities of medication administration, Au nanoparticles, NIR laser, and radiotherapy are combined to form quadrapeutics. This therapeutic approach has been demonstrated to boost the efficacy of basic chemotherapy by more than 17 times while utilizing only approximately 3%–6% of the clinical features doses of chemotherapeutics and radioactivity, without provoking adverse effects or residual tumors, *in-vivo* studies of a main and microscopic leftover HNSCC (Lukianova-Hleb and Lapotko, 2015). The efficiency of porphysome nanoparticles was evaluated for targeted photothermal ablative treatment and for improving fluorescence and photoacoustic images of head and neck cancers. According to the findings, porphysomes made it possible to image head and neck cancers and to specifically remove these tumors (Muhanna et al., 2015). Another strategy was devised to create a special Nano platforms made of gold nanoparticles encapsulated with cisplatin and glucose. This platform serves as both a radiosensitizer and a drug carrier, delivering cisplatin to the tA431 HNSCC tumor cells only. This nano platform showed effective tumor imaging detection capabilities (Davidi et al., 2018). The researcher investigated a non-invasive technology-focused technique to address certain current issues with OSCC detection, where a pathology biopsy remains the prevailing gold standard up to this point. This research team created nano-graphene oxide nanoparticles (NGOs) paired with AF750-6Ahx-Sta-BBN (specific PRGR peptide) via π and hydrogen bond, which resulted in NGO-BBN-AF750; a test with immunofluorescence functionality by taking benefit of the reality that peptide-releasing gastrin receptor (PRGR) is upregulated in HNSCC and serves as a possible target for OSCC fluorescent imaging systems. As opposed to the peptide antagonist AF750-6Ahx-Sta-BBN, NGO-BBN-AF750 demonstrated strong cellular internalization capacities. Generally, this investigation gives insights into an NGO nano colloids-based nano-probe for PRGR-based targeting near-infrared fluorescence for OSCC. Nano-based targetable approaches have presented a promising candidate for the selective distribution of a broad range of therapeutic molecules (Li et al., 2020).

### 6.9 Drug and gene-delivery vectors

Another active area of research is the development of nano-vectors for drug and gene delivery. The goal in drug delivery is to achieve a higher concentration of anti-tumor drugs at the tumor site since anti-tumor drugs that target tumor cells can damage normal cells. Despite the development of effective drugs, it remains a challenge to deliver them exactly where they are needed. Nanovectors are promising because they can cross the blood-brain barrier and have improved permeability and retention. Gold nanoparticles are widely used because of their improved retention and permeability properties, which facilitate their accumulation in tumors. However, the physical properties of gold nanoparticles (size, surface charge) as well as the route of administration are related to their efficacy (injection methods, dosage). Thus, Nasseri et al. investigated the correlation between the accumulation efficiency and physical properties of nanoparticles and dosing methods. They applied single or multiple dosing methods to deliver PEGylated gold nanoshells (GNS) and gold nanorods (GNR) to tumors, and neutron activation analysis was used to quantify the amount of gold present in the tumor and liver. According to their findings, multiple administration is preferable to single administration for both GNS and GNR accumulation in tumors, and small GNR accumulates in tumors in higher amounts than large GNR [205]. In this case, PEGylation is very important because this type of passive agent can prevent the reticuloendothelial system from getting rid of it in a non-specific manner (Chen et al., 2017). In addition, gene transfer provides an effective tool for the treatment of head and neck cancer, especially when siRNA interference techniques are used. One of the most popular methods of gene delivery is the gene gun. Mandatory gene delivery into the target tissue uses gold particles coated with a thin layer of DNA or siRNA. To stimulate immune responses against malignancies in normal organs, particles can be injected with DNA vaccination (Eusébio et al., 2021). Tumors can also receive genetic material. For example, transferrin or tumor-targeted peptides are used to enhance the targeting and uptake of cationic liposomes for HNSCCs (Jiang Z. et al., 2019).

### 6.10 Radiation enhancers

Due to their strong absorption capacity, gold nanoparticles are excellent radiation enhancers. In malignancies, gold nanoparticles can increase the limited radiation dose by over 200% [209]. Compared to the untreated group, mice with tumors treated with gold nanoparticles have a 1-year survival rate of nearly 86% (Chen et al., 2020). Radiation efficacy can also be improved by using titanium nanoparticles. Doping these titanium nanoparticles with rare Earth elements such as lanthanides and gadolinium can make them even more effective (Song et al., 2022).

Numerous strategies have been developed using nanoscience to improve radiation as an HNC drug. Studies on the use of nanoparticles to improve radiation therapy for HNC are still at an early stage. In the treatment of extremely invasive and radioresistant squamous cell carcinoma of the head and neck, a recent study investigated the use of gold nanoparticles to improve radiotherapy (nanogold radiotherapy) (SCCVII). After intravenous administration of Au nanoparticles, 12 rats with subcutaneous SCCVII tumors were exposed to superconducting Wiggler beam (42 Gy, 68 keV) for one minute. The administered nanostructures, 1.9 nm in diameter, contained gold centers (commercially available as AuroVistTM). After the rats received the nanoparticles for an extended period of time (>200 days), 67% of the rats survived, but only 25% of the rats that did not receive the nanoparticles survived (Hainfeld et al., 2010). Another study investigated how radiation absorption can be increased by using Au nanoparticles targeting cetuximab to combat tumor radioresistance. To this end, 36 mice were subcutaneously injected with HNSCC cells and the tumor mass of each animal was determined. Once the tumor reached a size of 8–10 mm, the mice were divided into six groups: Group 1 served as control, Group 2 received radiation as sole therapy, Group 3 received cetuximab (CTX) alone, Group 4 received radiation plus CTX, Group 5 received radiation plus IgG-coated (non-targeted) Au nanoparticles, and Group 6 received radiation + CTX-coated (targeted) Au nanoparticles. Tumor development in groups 1–5 progressed over time. The fact that disease did not worsen or progress in group 6 (irradiation plus CTX-Au nanoparticles) suggests that CTX-Au nanoparticles significantly improved tumor radiosensitivity compared with previous treatment regimens (Popovtzer et al., 2016).

A different method was used to use GBNs as radiosensitizers, although the basic process is not fully understood due to conflicting *in vivo* results. In this study, HNSCC cells received AGuIX^®^ before being exposed to radiation. Their endocytosis and lysosomal aggregation as a result of this therapy resulted in complicated DNA breaks that alone triggered intracellular ROS generation upon radiation exposure, eventually leading to the death of these cell lines and autophagy. Further exacerbation of these combined effects was caused by autophagy/autophagic cell death; however, the exact mechanism is still unclear (Simonet et al., 2020). In addition, hafnium nanoparticles are currently being investigated in phase I/II clinical trials for radiation enhancement (NBTXR3) in the treatment of HNSCC and other cancers. The hafnium oxide nanoparticle NBTXR3 is a radioactive particle. It enhances targeted death of cancer cells when exposed to radiation (Bonvalot et al., 2019).

miRNAs were examined for overall expression in 43 tumor samples, and a group of chosen miRNAs were checked in a separate collection of 51 tumors. The study found that patients with short-term and long-term locoregional management of their condition had distinct expression levels of miR-15b-5p (LRC). In contrast to patients with low miR-15b-5p expression, HNSCC patients with higher expression have significantly longer locoregional recurrence-free survival, according to Kaplan-Meier analysis. Finally, miR-15b-5p is an independent prognostic biomarker of LRC in HNSCC individuals, according to multivariable Cox regression analysis (HR = 0.25; 95% CI = 0.05–0.78; p0.016). In addition, miR-15b-5p represents a potentially useful biomarker for individualized treatment of patients with HNSCC (Ahmad et al., 2019).

### 6.11 Nanovaccines

Recognition of tumor-specific antigens, concurrent administration of adjuvants, and the delivery vehicle are critical to the efficacy of therapeutic cancer vaccines. In HNSCC, certain antigens like p53 and CSC-related proteins, and antigens like HR-HPV oncogenic proteins might prepare immune cells to elicit a potent immune action (Sun et al., 2022). For instance, immunization directed against HR-HPV E6 and E7 oncoproteins can result in a full histologic response and T-cell reaction against HPV-16 (van Poelgeest et al., 2016). Patients with HNSCC have demonstrated modest vaccine-specific immunity following adjuvant DC-based immunization against p53 (Wefers et al., 2018). The technical assistance, time, and financial constraints associated with neoantigen identification and vaccine manufacture are significant constraints due to the assertive nature and brief life duration of individuals with recurring or metastatic HPV-HNSCC. However, for patients with HNSCC, HPV-HNSCC vaccines are a promising approach to prevent cancer recurrence and de-escalate therapy. Other methods, such as those that specifically target stem cell transcription factors like NANOG, could destroy CSCs (Wefers et al., 2018). Moreover, their clinical therapeutic efficacy in treating advanced HNSCC may be limited by the immunosuppressive components in the tumor microenvironment and the consecutive physical spatiotemporal features (Sun et al., 2022). Nanomedicine has the ability to enhance the anticancer effects of vaccines and reverse immunosuppression. In addition to direct delivery of tumor antigens and adjuvants to lymphoid tissues, nano-vaccines have the potential to increase treatment efficacy by containing immunosuppressive antagonists or immunostimulatory agents (Grippin et al., 2017). This is conceivable because medicinal chemicals may be arbitrarily selected and combined on nanocarriers based on the desired usage.

## 7 Management of HNSCC with miRNA-based nanomedicine

According to recent studies, the tumor microenvironment in HNSCC can control the tumor response to radiation treatment through a cross-talk between the cancer cells and the cancer microenvironment (Gurin et al., 2020). Accordingly, miR-9, a component of the ligand-activated nuclear receptor complex associated with inflammation and innate immunity, was found to be elevated in HPV-positive HNSCC exosomes and to enter macrophages, where it leads toM1 macrophage polarization. High levels of miR-9 made HPV-positive HNSCC more susceptible to radiation, and subsequent survival analysis revealed that miR-9 levels were positively connected with a better prognosis (Tong et al., 2020). Several miRNAs have been reported as biomarkers for therapy monitoring in human HNSCC biofluids. For example, in the plasma of patients with HNSCC, increased expression of miR-186-5p, miR-142-3p, miR-195-5p, miR-574-3p, and miR-374b-5p was connected with a worse outcome and offers promising markers for prognosis and therapeutic monitoring (Summerer et al., 2015). Interestingly, ten HNSCC- or associated with radiation miRNAs (miR-93, miR-142-3p, miR-125a, miR-200a, miR-213, miR-203, let- 7a, let-7b, let-7g, and let-7i) were found in saliva samples. Additionally, miR-93 and miR-200a were considerably more articulated among them 12 months after radiation therapy than they were at baseline. Therefore, these miRNAs appear to be suitable candidates for use as biomarkers in post-radiation therapy monitoring in HNSCC (Greither et al., 2017). Recent research revealed that several miRNAs were altered in OSCC compared to healthy adjacent tissues. It was investigated whether the detected miRNAs were also in the blood of the same patients. 30 miRNAs were detected in the patient’s serum, in addition to the 48 miRNAs that were differentially transferred in the tissue. Compared with normal tissue, tumors have significantly higher levels of miR-32-5p, and studies of its upregulation in serum have also been performed. As a result, the study suggests a verified miRNA profile that could be utilized as a possible non-invasive biomarker of OSCC (Schneider et al., 2018). There have been several investigations on HNSCC that use lipid-based technologies. Piao *et al.* prepared cationic lipid nanoparticles from alpha-Tocopheryl polyethylene glycol 1,000 succinate, cholesterol, and dimethyl octadecyl ammonium bromide (DDAB) for pre-miR-107 administration after showing that miR-107 (miR-107) was considerably dysregulated in HNSCC tumors in contrast to healthy cell regions (Piao et al., 2012). Compared with infusion of free pre-miR-107, nanoparticle-mediated pre-miR-107 delivery increased pre-miR-107 levels in HNSCC cells. Moreover, pre-miR-107 suppressed tumor cell viability, invasion, and motility in the existence of nanoparticles. In addition, the ^188^Re liposome was found to upregulate the anti-tumor let-7 miRNA (Lin et al., 2016a). In a work, by Lo *et al.*, several peptides were formed as nanovectors for the transport of irinotecan and miR-200 to increase tumor-specific deposition. Liposomes and solid lipid nanoparticles (SLN) were altered using a pH-sensitive, self-destructive PEG shell. Genes linked to EMT are regulated and suppressed by the microRNA miR-200. As a topoisomerase I inhibitor, irinotecan hinders the remodeling of double-stranded DNA, which results in cell apoptosis. In this research, it was shown that the cleavable PEG layer was responsive to lower extracellular pH while the targeted peptides attached to the nanoparticles facilitated the reabsorption and liberation of miR-200 and irinotecan onto HNC SAS cells. In a mouse model of a SAS tumor, Onivyde and other compositions led to different results. This combination treatment, on the other hand, caused SAS-treated cells to die more quickly and had better clinical efficacy and tolerability. As a result, this work has demonstrated how combined treatment with pH-sensitive coatings and altered nanoparticle targeting can be a cutting-edge method for treating HNC (Lo et al., 2020). The ideal nanocarrier should protect the miRNA and the therapeutic agent from the circulatory environment and efficiently deliver the therapeutic agents to tumor cells, and it is extremely important to investigate their safety profiles *in vivo*, with particular attention to their toxicity and immune response. It was found that the tumor suppressive let-7 miRNA could be upregulated by ^188^Re-liposome and involved in mediating the therapeutic efficacy of this radiopharmaceutical novel strategy for targeting HNSCC partially via induction of *let-7* microRNA (Lin et al., 2016b). Additional studies showed that a unique synthetic miR-30a-5p mimic nanomedicine might block the growth receptor production and slow tumor development in HNSCC xenograft tumor concepts without causing any adverse side effects. Further research identified the miR-30-5p group as a tumor suppressor and possible therapeutic strategy in HNSCC subgroups and showed that miR-30a-5p slowed HNSCC cell motility and inhibits EGF-induced penetration *in-vitro.* Reduced miR-30 family production was also associated with DNA replica loss, clinical prognosis, and promoter hypermethylation (Saleh et al., 2019).

## 8 Ongoing and completed clinical trial for the management of HNSCC

The data from ongoing and finished clinical studies including the use of NMs in HNC are presented in Table 5.

**TABLE 5:** Distinctive nanomedicine is being used in clinical studies for HNC.

Product	Dosage form	Drug delivery	Trial phase	Status	NCT number	Main findings	Locations	Ref
Abraxane	Nanoparticle	Paclitaxel	Phase 1/2	Completed	NCT00851877, NCT00833261	All-cause mortality rate is 2.94%, and serious adverse events is 8.82% (with only one event of blood and lymphatic system disorder)	The University of Texas Southwestern Medical Center, Dallas, Texas, United States (U.S); Baylor Research Institute, Dallas, Texas, U.S	Adkins et al. (2021), Oppelt et al. (2021)
			Phase 2	Recruiting	NCT01412229, NCT00736619	All-cause mortality rate is 0%, and serious adverse events is 63.64% (minor lymphopenia, leukopenia, neutropenia, anemia, and dermatitis)	The University of Texas Southwestern Medical Center, Dallas, Texas, United States; Baylor Research Institute, Dallas, Texas, United States; Medical College of Wisconsin, U.S	
			Phase 2	Active, not recruiting	NCT04857164, NCT04922450 NCT02270814, NCT03174275	Serious adverse events are 33.3% (minor cardiac disorders, gastritis, vomiting, hepatobiliary disorder, lung, skin, and urinary infection)	University of Washington, United States; Vanderbilt University, Tennessee, U.S	
Albumin-bound rapamycin NPs	Nanoparticle	Rapamycin	Early phase 1	Completed	NCT02646319	Findings not mentioned	Memorial Sloan Kettering Cancer Center, New Jersey, U.S	Abu-Khalaf et al. (2015)
Anti-EGFR immunoliposomes	Liposome	Doxorubicin	Phase 1	Completed	NCT01702129	Findings not mentioned	National Cancer Centre/Cancer Hospital, Beijing, China	Mamot et al. (2012)
AuroShell	Gold nanoshell nanoparticle	Gold-Silica nanoshells	Not applicable	Completed	NCT00848042	Findings not mentioned	Hunan Cancer Hospital, Hunan, China	Cheng et al. (2020)
BIND014	Nanoparticles	Docetaxel	Phase 1	Completed	NCT01300533	Findings not mentioned	University of Mississippi Medical Center, Mississippi, United States; University of Arkansas for Medical Sciences, Arkansas, U.S	Najjar et al. (2017)
CMP-001	Virus-like particle	TLR9 agonist	Phase 2	Recruiting	NCT04633278	Findings not mentioned	Vanderbilt University, Tennessee, U.S	Cheng et al. (2020)
Dimethylaminoethane-carbamoyl (DC)-cholesterol liposome	Liposome	EGFR antisense DNA	Phase 1	Completed	NCT00009841	Findings not mentioned	Mayo Clinic Rochester, Minnesota, United States; Mayo Clinic in Arizona, Arizona, U.S	Cheng et al. (2020)
Doxil	Lipid sphere (Liposome)	Doxorubicin	Phase 1	Completed	NCT00252889, NCT02262455, NCT04244552	Findings not mentioned	University Hospital Basel, Switzerland	Rose et al. (2020)
LiPlaCis	Liposome	Cisplatin	Phase 2	Completed	NCT01702129	From all the three Auroshell (3.5, 4.5, 5), the Auroshell 5 is showing some serious adverse events of 40% (general disorder like numbness, cardiac event and neoplasm)	Baylor College of Medicine Houston, Texas, U.S	Mamot et al. (2012)
Mitoxantrone hydrochloride liposome	Liposome	Mitoxantrone hydrochloride	Phase 1	Not yet recruiting	NCT04902027	Findings not mentioned	Los Angeles, California, United States; Greenbrae, California, U.S	Cheng et al. (2020)
NBTXR3	Nanoparticle	HfO_2_-containing NPs	Phase 3	Recruiting	NCT04892173	Findings not mentioned	University of California-San Diego, La Jolla, California, United States; University of Arkansas for Medical Sciences, Arkansas, U.S	Zhang et al. (2020a)
NC-6004	Nanomicelles	Cisplatin	Phase 1/2	Completed	NCT03109158	Findings not mentioned	University of Pittsburgh Cancer Institute, Pennsylvania, U.S	Subbiah et al. (2018)
ONM-100	Nanoparticle	FDA-approved fluorophore	Phase 2	Completed	NCT03735680	Findings not mentioned	Christiana Care Health Services, Delaware, U.S	Voskuil et al. (2020)
PRECIOUS-01	Nanoparticle	Threitolceramide-6; NY-ESO-1 peptides	Phase 1	Recruiting	NCT04751786	Findings not mentioned	Sarah Cannon Research Institute, Nashville, Tennessee, United States; Memorial Sloan Kettering Cancer Center, New York, U.S	Creemers et al. (2021)
SNB-101	Nanoparticle	Irinotecan	Phase 1	Recruiting	NCT04640480	Findings not mentioned	City of Hope, California, United States; Mayo Clinic Phoenix, Arizona, U.S	Hu et al. (2021)
TumoCure	Polymer-based gel	Cisplatin	Phase 1	Not yet recruiting	NCT05200650	Findings not mentioned	Herlev and Gentofte Hospital, Herlev, Dermark	Subbiah et al. (2018)

## 9 miRNAs as emerging novel therapeutic targets for HNSCC

In contrast to their use in research and analysis, miRNAs are good therapeutics (depending on the type of mRNA they alter) (Lajer et al., 2012). miRNAs are easy to handle because they interact with many other molecules to alter many different physical processes (Cataldo et al., 2016). A new method to control oncogenic and tumor suppressive pathways include synthetically produced “miRNA sponges,” miR inhibitors (antimiRNAs oligonucleotides), miR antagonists (antagomiRs), and miR mimics (agomiRs) (Tseng et al., 2017). Each phase of the cell cycle is regulated by microRNAs and finding out how their altered expression is expressed could be useful for developing new drugs and therapeutic options. The abduction of miR-122 observed in an initial report on the innovative treatment of hepatitis C virus (HCV) patients by miravirsen (an anti-miR-122 oligonucleotide), currently, in phase 2a clinical trials, is one type of evidence. HCV RNA Levels decreased in a dose-dependent pattern as a result of this treatment. Despite the differences between cancer types, this study provides much hope for the use of miRNA therapies in a variety of malignancies. Other miRNA therapies are in preclinical and phase 1 development. It is expected that these methods will be popularized beyond a simple description. Even though they have not been tested in people yet, some miRNAs would be great for these treatments. Since cancer cells are usually unable to induce programmed cell death, the apoptotic mechanism is one of the most important ways to develop new cancer treatments (Irshad et al., 2018). Thus, any potent drug that accelerates apoptosis can directly or indirectly stop cancer growth. miR-99a analogs induce apoptotic signaling pathways in the tongue SCC cell line, which slows down the growth of cells (Sun and Yan, 2021). Moreover, miR-31pp inhibitor reduced cell death in oral mucosal cancer cell lines (Solomon and Radhakrishnan, 2020). Moreover, miR-100 restoration reduced cell migration and proliferation by increasing apoptosis in HNSCC cell lines. Similarly, miR1e-transfected cancer cells showed marked cell cycle alteration and more cell death. In contrast, the traditional oncogenic miR-21 was shown to suppress apoptosis (Yuan et al., 2017). These are only a handful of known cases. Numerous other functionally studied miRNAs in HNSCCs are either actively or passively associated with apoptotic metabolic pathways, opening up a variety of new opportunities for effective therapeutics. Since miRNAs can show how well a patient does with radiotherapy or chemotherapy, they can be used to help HNSCC patients in a specific way.

In addition, it was recently discovered that miRNAs can alter chemoresistance and radiosensitivity. For example, let-7 inhibits the spread of cancer by reducing susceptible cells and suppressing chemoresistance (Ma et al., 2021). Similarly, miR-21, which is involved in the control of the NANOG/STAT3 pathway, may prove to be a therapeutic key to overcoming HA/CD44-induced chemoresistance and apoptosis in HNC cells. Moreover, by suppressing the expression of HMGA2, transfection of pre-miR-98 in HNSCC cell lines increased resistance to cisplatin and doxorubicin (Li et al., 2019).

But miRNAs cannot be used alone to develop a drug distribution strategy that works and delivers drugs to the right sites. Therapeutic RNA can leave the circulatory system, cross the plasma membrane, bypass endosomal vesicles, and penetrate the cell to reach specific areas. In addition, immune cells (7–20 kDa) or the kidney (50 kDa) are responsible for the clearance of non-conjugated medicinal RNA molecules (Falzone et al., 2019). The distribution of synthesized double-stranded hairpins extracellularly by polymerization with proteins or lipids can be used to alter or decrease the concentrations of mature miRNAs. Considering that miR-34a administration suppressed cellular proliferation and apoptosis in two carcinoma cell lines (colon) and simulated lung metastasis of mouse carcinomas, miR-34a treatment may be beneficial in HNSCC cells (Liu et al., 2020).

In cases where local delivery is possible, the use of unmodified dsRNAs *in-vivo* is of minimal benefit because they are likely to be degraded by nuclease. A viral vector containing polymerase III promoters for steady miR recovery can be used to induce high transcription of these miRNAs from well-specified transcription starts and complementary regions, although they are not cell-specific. On the other hand, RNA polymerase II promoters can be used to produce miRNAs for a tissue-specific strategy or ectopic miRNA production (Li Y. et al., 2022). In HNSCC, subsequent approaches, such as miR-375, can be used to effectively restore downregulated miRNAs.

In addition, using a viral system to reintroduce miRNAs carries a number of dangers. One of the main dangers is that the introduced compounds can integrate into the host DNA at an unanticipated site, triggering a proto-oncogene and causing insertional mutagenesis. There are also a lot of problems with these methods. For example, releasing vectors into the body of the host can cause a strong immune response, and retroviral vectors can only be released into cells that are actively dividing (Sayed et al., 2022).

Currently, host miRNAs are often silenced using antagomiRs (recently discovered chemically engineered oligonucleotides) (Li Y. et al., 2022). When antagomir-155 was introduced into hairless mice, the effect of overexpression of miRNAs was reversed in HNSCC, resulting in decreased cellular proliferation and increased induction of apoptosis in KB cells transfected with miRNA-155. To enhance the efficacy of these antagomiRs when administered intravenously, they were also complexed with interfering nanoparticles (iNOP), a newly discovered molecule. In mice, systemic administration of iNop cross-linked with antimir-122 suppressed miRNA-122 without eliciting an immune response (Zeshan et al., 2016). Another way to silence miRNAs is to use chemically modified anti-miRNA oligonucleotides (AMOs), which attach very effectively and precisely to the targeted RNA. When miRNA-21 was inhibited with AMO, increased apoptosis, decreased survival, and increased proliferation occurred in tongue SCC cell lines. Moreover, miR-21AMO doses repeatedly caused severe apoptosis and reduced cell proliferation in hairless mice, which eventually suppressed tumor growth (Wang et al., 2017).

Onco-miRNAs can also be inhibited in HNSCC by using miRNA sponges or miRNA masks. By producing an mRNA with multiple tandem binding domains for the comprehensive group of certain miRNAs, miRNA sponges prevent some miRNAs from attaching to specific targets and exerting an effect. However, the miRNAs-masking antisense oligonucleotide technique, which consists of corresponding antisense oligonucleotides at the miRNA interaction site in the untranslated region of a targeted mRNA, shows its effect by disrupting the relationship between specific miR-mRNA pairings (Li Y. et al., 2022). Due to the sequential targeting of numerous pathways, this technology offers an advantage over others in that there are fewer off-target or undesirable consequences. In addition, miRNAs could increase radiosensitivity (Jurkovicova et al., 2022). By restoring the production of tumor-suppressive miRNAs, certain epigenetic treatments may also lead to the return of uneven methylation or acetylation of tumor tissue. Most of the approaches discussed above are still in the experimental phase. Since multiple miRNAs and various miRNA interactions with the transcriptome have been implicated in cancer development, our potential strategy should focus on disrupted miRNA systems. To achieve this goal, it is necessary to target miRNA biogenesis devices or regulatory pathways. However, it is strongly advisable to explore the full power of these drugs (Li Y. et al., 2022).

## 10 Challenges and opportunities in the application of miRNA in HNSCC

The most common causes of death in people with HNSCC are localized recurrence and metastasis. Therefore, it is crucial to find biomarkers such as miR-16, miR-93, miR-141, miR-150, miR-1323, miR-18b, miR-28-5p, miR-301a, and miR-371a-5p for earlier diagnosis of localized recurrence and to increase the survival rate of patients with HNSCC. The initial stage the of metastatic phase is migratory and invasive. During the cycle of epithelial-mesenchymal transition (EMT), epithelial cancer cells become migratory mesenchymal cells, which can either generate new tumors in the same area (recurrence) or in a different location (metastasis) (Yang et al., 2019). Because of this, the expression of mesenchymal biomarkers like fibronectin, N-cadherin, and vimentin goes up during this phase, while the expression of epithelial biomarkers like E-cadherin goes down (Das V. et al., 2019). Several transcriptional regulators are involved in the EMT process, including Twist, Slug, ZEB1, Snail, and ZEB2, whose expression profiles are correlated with the presence of metastatic disease. miRNAs are involved in the regulation of recurrence, EMT, and invasion, as shown by several recent research (Feng et al., 2022). Improper binding of miRNAs is one of the fundamental challenges in their study. For example, the Miranda algorithm suggests 1,130 targets for the single miRNA, *i.e.*, miR-21, while other approaches, such as target scan, estimate 186 and 175 targets. By searching for such miRNAs that could modulate growth when translated into cell lines, researchers aimed to discover important miRNAs rather than examining a broad list of likely downstream targets (Subha et al., 2022).

The lack of high-quality tumor samples for expression evaluation is a significant obstacle in the study of miRNA biomarkers. For such extraction of high-quality RNA from fresh-frozen tumors, miRNA expression evaluation is often performed. However, since most tumors are routinely stored in formalin-fixed-paraffin embedded (FFPE), the RNA in archived tumors is severely damaged. It was difficult to collect a large number of frozen tumors for a miRNA profiling study, especially since the focus was on oropharyngeal SCC. Instead, it was demonstrated that preserved FFPE tumor samples obtained according to accepted clinical procedures were used. A brand-new PCR-based profiling technique for measuring miRNA expression was developed to overcome the difficulty of evaluating low-quality RNA. This technique performs well in terms of detection accuracy and specificity even when used with FFPE tumors [258]. Another potential problem is obtaining profiles with unquestionable specificity that leave no doubt about the outcome of the diagnostic method due to their uniqueness for a particular disease. If some miRNA types are overexpressed simultaneously in different diseases, such as liver fibrosis and hepatitis B infection, this could lead to misdiagnosis (Kabzinski et al., 2021).

In addition, taking medications during chronic hepatitis C therapy affects this value. With this in mind, the industry will not be able to adopt a broader range of diagnostic tools until standardized procedures are in place. For the treatment of patients with chronic HNSCC who do not respond to platinum-based chemotherapy, the anti-PD-1 immunotherapeutics nivolumab and pembrolizumab were approved in 2016 (von der Grün et al., 2019). Immunotherapy is a modern way to treat HNSCC. The goal of this treatment is to make the immune system better at killing cancer cells. Because of this, it is very important to find biomarkers that can predict who might benefit from immunotherapy. Circulating miRNAs have been shown to be able to predict response to anti PD-1/PD-L1 therapy in a variety of cancers (Liu et al., 2019; Pesce et al., 2020). Recently, let-7 group expression was found to be significantly downregulated in HNSCC, with PD -L1 expression showing a negative correlation. Moreover, *in vivo* studies have shown that upregulation of Let-7a/b improves immunotherapy in HNSCC by enhancing antitumor immunotherapy with a CD152 antibody. The involvement of miRNAs in signaling pathways related to oncogenesis, metastasis, and treatment resistance of HNSCC is becoming increasingly clear. miRNAs may be valuable for the initial phase of a tumor or even its relapse, as well as for tailored therapeutic regimens, according to a series of *in vitro* and *in vivo* results compiled by numerous experts. Even though there have been a lot of peer-reviewed scientific studies, there is still a lot of uncertainty about how well biomarkers based on miRNA can be used to find and treat HNSCC. There are cases where a number of *in-vitro* results are inconclusive, potentially leading to conflicting results between studies. Therefore, there is an increasing need for additional predictive studies to promote basic and integrative work. However, integrating these different miRNA signatures as biomarkers would allow for more personalized approach to the clinical management of HNSCC patients, and ultimately improve clinical outcomes (Yu et al., 2019).

## 11 Conclusion

HNC is one of the most common cancers in the world and is expected to increase in the coming years. Due to drug targeting challenges, there is a critical need for inventive techniques in cancer therapy, as chemotherapy of HNSCC is apprehensive with problems (comorbid diseases, reduced organ activity). The development of nanocarriers that can bypass the immune response and that can be directed to the tumor through various targeting mechanisms has made nanomedicine (nanoparticles) an effective approach to achieve these goals and they could also increase the efficacy of chemotherapy while reducing its cytotoxicity to normal tissues. These nanocarriers make it possible for anticancer drugs to be delivered at the same time, which helps combat tumor resistance even more. In addition to intervention, nanomaterials can also change the way HNSCC is treated by using diagnostic techniques that enable simultaneous detection and treatment. Nanocarriers need more study to find out about their cytotoxicity, biocompatibility, long-term effects, and limits. One novel approach is by the use of miRNA.

The miRNA-based treatment is based on the idea that diseases alter the miRNA profile and that this can be corrected by restoring the downregulated or upregulated miRNA to its baseline level. The level of miRNA will aid in the prediction and early diagnosis of HNSCC. miRNA is therefore proposed and explored as a helpful and prospective biomarker and therapy for HNSCC. These miRNAs are also having certain limitations like biological instability, short half-life in the bloodstream, limited intracellular delivery, and off-target effects with associated toxicity, additional problems arise in miRNA-based treatment and distribution. To overcome these limitations, nanotechnology appears to be an excellent alternative to conventional approaches for miRNA detection and a potential delivery system for the efficient distribution of miRNA. The miRNA can be coupled with nanocarriers in order to increase the resistance of oligonucleotides to nuclease degradation, reduce the possibility of immunological rejection, and improve cellular absorption, oligonucleotides (such as siRNA, anti-miRNA, and miRNA). A variety of nanocarriers, including lipid-based nanocarriers, metallic nanoparticles, dendrimers, polymeric nanoparticles, and carbon nanostructures, can be used for the prognosis and therapy of HNSCC. Nanomaterials (NMs) are excellent carriers for radiosensitizers, drugs, genes, vaccines, and photosensitizers. Though targeted delivery, controlled release, sensitivity to stimuli, and distribution of various drugs could allow NMs to improve the evaluation, treatment and outcome of HNSCC patients.

The review provides a summary of ongoing and completed clinical trials with NMs in HNSCC. Most nanocarriers technologies can selectively embed tumors through damaged tumor micro vessels, which are administered intravenously and systemically to treat cancer. Improved permeability and retention are still not understood, and the biosafety of NMs is still a challenge. Finally, recent advances in research on NMs have opened a variety of new opportunities for the treatment and detection of HNSCC. Accelerated advances in tumor research, nanotechnology, and the development of NMs that combine the advantages of nanotechnology with the physiological properties of HNSCC have been made possible by expanded multidisciplinary collaboration. As a result, experts expect that the use of NMs will improve the prognosis for people with HNSCC. The emergence of nanotechnology in the development of drug delivery systems has proven to be an important avenue for achieving novel methods and greater safety and efficacy than conventional cancer treatments.

## Figures and Tables

**Table:** 

**PEG -P[Gu]**	Poly(ethylene glycol)-poly(glutamic acid)
**Akt**	Protein kinase B
**ANK**	Ankyrin repeats
**APC**	Adenomatous polyposis coli
**AREG**	Amphiregulin
**ATM**	Ataxia telangiectasia mutated
**ATR**	Ataxia-telangiectasia and Rad3 related
**Bax**	B-cell lymphoma associated X protein
**BCl-2**	B-cell lymphoma
**BLM**	Bleomycin
**BRCA1**	Breast cancer type 1 gene
**CA9**	Carbonic anhydrase 9
**CDF**	Curcumin-difluorinated
**c-Met**	Mesenchymal-epithelial transition factor
**CSCs**	Cancer stem cells
**CTCs**	circulating tumor cells
**ctDNA**	Circulating tumor DNA
**DDAB**	Dimethyl octadecyl ammonium bromide
**DDP**	Cisplatin
**DL**	Delta-like
**EBV**	Epstein-Barr virus
**ECD**	Extracellular domain
**EGFR**	Epidermal growth factor receptor
**EMT**	Epithelial-mesenchymal transition EMT
**ERK**	Extracellular signal-regulated kinase
**EVs**	extracellular vesicles
**FA**	Folate
**FBXW7**	F-Box and WD repeat domain containing
**FGF2**	Fibroblast growth factor-2
**FR**	Folate receptor
**Gab1**	Growth factor receptor bound protein 2 associated binder 1
**GLUT1**	Glucose transporter 1
**GNR**	Gold nanorods
**GNS**	Gold nanoshells
**GSK-3β**	Glycogen synthase kinase-3-β
**HCV**	Hepatitis C virus
**HGF**	Hepatocyte growth factor
**HIF-1α**	Hypoxia-inducible factor-1α
**HIOEC**	Human immortalized oral epithelial cells
**HMGA2**	High mobility group protein 2
**HNSCCs**	Head and neck squamous cell carcinomas
**HPV**	Human papilloma virus
**HT**	Hyperthermia
**IGF-1R**	Insulin-like growth factor-1 receptor
**iNOP**	Interfering nanoparticles
**IRS-1**	Insulin receptor substrate-1
**JAG**	Jagged
**JAK**	Janus kinase
**KRAS**	Kirsten rat sarcoma 3
**Leuk1**	Precancerous leukoplakia
**Ma**	Macrophages
**MAPK**	Mitogen-activated protein kinase
**MDC1**	Mediator of DNA damage checkpoint 1
**MiRNAs**	MicroRNAs
**MNPs**	Superparamagnetic nanoparticles
**MOFs**	Metal-organic frameworks
**MRI**	Magnetic resonance imaging
**MSV**	Multi-stage vector
**mTOR**	Mammalian target of rapamycin
**NGOs**	Non-graphene oxide nanoparticles
**NGS**	Next-generation sequencing
**NICD**	NOTCH intracellular domain
**NIR**	Near-infrared
**NLCs**	Nanostructured lipid carriers
**NM**	Nanomedicine
**NMs**	Nanomaterials
**NOCTH**	Neurogenic locus notch homolog protein
**NS**	Normal saline
**OSCC**	Oral squamous cell carcinoma
**p53**	Tumor protein p53
**p53BP1**	p53 binding protein 1
**p63**	Tumor protein p63
**PAA**	Polyacrylic acid
**PDK1**	Phosphoinositide-dependent protein kinase 1
**PDT**	Photodynamic treatment
**PEG**	Polyethylene glycol
**PGK**	Phosphoglycerate kinase
**PHM**	Pleckstrin homology motif
**PI3K**	Phosphatidylinositol 3-kinase
**PIP2**	Phosphatidylinositol 4,5-bisphosphate
**PIP3**	Phosphatidylinositol 3,4,5-triphosphate
**PPTT**	Plasmonic photothermal therapy
**PRGR**	Peptide-releasing gastrin receptor
**PTEN**	Phosphatase and tensin homolog
**PTKM**	Protein tyrosine kinase motif
**PTX**	Paclitaxel
**QDs**	Quantum dots
**RM**	Relapsed metastatic
**ROS**	Reactive oxygen species
**RT**	Radiotherapy
**SalB**	Salvianolic acid B
**SEMA**	Semaphorin
**SHP2**	SH2-containing protein tyrosine phosphatase
**SiAuNS**	Silica-gold nanoshells
**STAT3**	Signal transducers and activators of transcription-3
**TACE**	TNF-α converting enzyme
**TAFs**	Tumor-associated fibroblasts
**TAM**	Transcriptional activation motif
**TGF-β**	Transforming growth factor beta
**TNM**	Tumor nodal metastasis
**TYK**	Tyrosine kinase
**VEGF**	Vascular endothelial growth factor
**α-TOS**	alpha-tocopheryl succinate
